# Principles of Endocrine Regulation: Reconciling Tensions Between Robustness in Performance and Adaptation to Change

**DOI:** 10.3389/fendo.2022.825107

**Published:** 2022-06-09

**Authors:** Rudolf Hoermann, Mark J. Pekker, John E. M. Midgley, Rolf Larisch, Johannes W. Dietrich

**Affiliations:** ^1^ Department for Nuclear Medicine, Klinikum Lüdenscheid, Lüdenscheid, Germany; ^2^ Mathematical Sciences Department, University of Alabama, Huntsville, AL, United States; ^3^ Consultancy Division, North Lakes Clinical, Ilkley, United Kingdom; ^4^ Diabetes, Endocrinology and Metabolism Section, Department of Medicine I, St. Josef Hospital, Ruhr-University of Bochum, Bochum, Germany; ^5^ Diabetes Centre Bochum/Hattingen, Ruhr University of Bochum, Bochum, Germany; ^6^ Ruhr Center for Rare Diseases (CeSER), Ruhr University of Bochum and Witten/Herdecke University, Bochum, Germany

**Keywords:** homeostasis, allostasis, adaptation, endocrine regulation, mathematical model, hypothalamic-pituitary-thyroid axis, triiodothyronine

## Abstract

Endocrine regulation in the hypothalamic-pituitary-thyroid (HPT) axis is orchestrated by physiological circuits which integrate multiple internal and external influences. Essentially, it provides either of the two responses to overt biological challenges: to defend the homeostatic range of a target hormone or adapt it to changing environmental conditions. Under certain conditions, such flexibility may exceed the capability of a simple feedback control loop, rather requiring more intricate networks of communication between the system’s components. A new minimal mathematical model, in the form of a parametrized nonlinear dynamical system, is here formulated as a proof-of-concept to elucidate the principles of the HPT axis regulation. In particular, it allows uncovering mechanisms for the homeostasis of the key biologically active hormone free triiodothyronine (FT3). One mechanism supports the preservation of FT3 homeostasis, whilst the other is responsible for the adaptation of the homeostatic state to a new level. Together these allow optimum resilience in stressful situations. Preservation of FT3 homeostasis, despite changes in FT4 and TSH levels, is found to be an achievable system goal by joining elements of top-down and bottom-up regulation in a cascade of targeted feedforward and feedback loops. Simultaneously, the model accounts for the combination of properties regarded as essential to endocrine regulation, namely sensitivity, the anticipation of an adverse event, robustness, and adaptation. The model therefore offers fundamental theoretical insights into the effective system control of the HPT axis.

## Introduction

Living systems face many challenges to their well-being and threats to their survival. Despite continual challenges, they show a remarkable ability to cope in each situation with various types of stress to minimize harm. While dealing with changing environmental conditions arising outside the system, summarized under the unspecific term stressors, the system must protect its inner environment to maintain its state and integrity (1, 2). A vital system property, required to resist stress and maintain a stable, relatively constant internal environment is termed homeostasis (3, 4). Homeostasis is an emergent property of biochemical networks, not a fixed target value (5). Typically, maintenance of homeostasis can be achieved through specific mechanisms involving negative feedback loops (6, 7). While it is vital for living systems to maintain their internal state of homeostasis against stress, they must be equally able to adapt as needed to varying adverse conditions (8). This, however, may require the system to be flexible in moving away from its homeostatic state and adjusting to new demands (9). Positive feedback loops, unlike their negative counterparts, can both amplify a variable of interest, and shift the system from its initial homeostatic state to a new one (10, 11). These dual necessities describe two antagonistic system properties, robustness as a tendency to resist change by preserving its homeostatic state, and adaptation as an ability to adjust the homeostatic state as required. In the event of stress, any conflict between the two opposing requirements must be reconciled. The task is non-trivial because the system must decide which clues to act on and which one of the two antagonistic responses may be more beneficial. The choices are: either to withstand the stress in its current homeostatic level or to shift to another homeostatic level which may better suit the altered external conditions.

The efficient combination of mechanisms to either support the homeostasis or to opt for its adaptation supports the resilience of a living system by choosing the most efficient path available (12). In this regard, human-body systems, unlike many technical equivalents, require both top-down and bottom-up regulation for achieving the desired outcome (13, 14).

**Figure 1:**
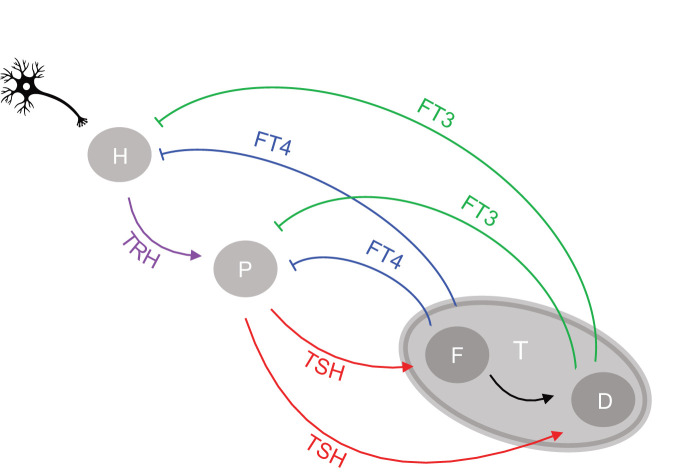
Network of the hypothalamic-pituitary-thyroid axis regulation. Afferent signals project to the hypothalamus from higher brain centers. The hypothalamus (H), pituitary (P), and thyroid gland (T) communicate through an interactive network of positive feedforward (arrows) and negative feedback loops (bars), involving as signals the respective hormones produced and released by each gland into the circulation, namely TRH by the hypothalamus, TSH by the pituitary and T4 and T3 by the thyroid gland. T3 is both synthesized *de novo* in the thyroid follicular cell (F) and converted from T4 as a precursor by deiodinases (D). The circulating free forms of T4 and T3 are referred to as FT4 and FT3, respectively.

The present study aims to elucidate further the underlying physiological mechanisms of the hypothalamus-pituitary-thyroid (HPT) axis regulation (15) (**Figure 1**). It is a proof-of-concept study that uses mathematical modelling as a tool to develop the conceptual framework. This allows the formulation of testable hypotheses. Specifically, we are interested in elucidating the mechanisms which account for the HPT responses to various challenges. To this end, we investigate the bidirectional exchange of information through the network. The thyroid system has to adapt not only to challenges arising from a shortage or excess of thyroid hormone supply but must also meet the energy and metabolic needs of the organism in non-thyroidal disease (16, 17). TSH secretion depends not only on the feedback of the supply of thyroid hormones but also on multiple other influences including genetic traits, allostatic load, psychosocial stress, and medication, all of which can alter the balance of the HPT axis (17). We propose that clinical population studies are not well suited to identify the underlying mechanisms of the HPT axis regulation (18). This is because their results may be compromised by their inability to take into account the different physiological mechanisms (18), which together determine the overall expression of free thyroxine (FT4) and free triiodothyronine (FT3) levels. One such mechanism may support the preservation of homeostasis, and the other may be responsible for an adaptation of the homeostatic state to a new one, thus providing optimum resilience to the system in stressful situations.

We next introduce important system properties. Under normal conditions the system will attempt to preserve its current optimal homeostasis by keeping the homeostatic variable in a tight range. In various pathological situations, on the other hand, only an orchestrated adaptation from the original homeostatic optimum may allow appropriate adjustment to new conditions (8). The adaptive response of the feedback loop to stress, which involves changing its homeostatic state, has been termed allostasis by some authors (8). As is well known, non-linear system behavior may cause range-dependent sensitivities (19). Consequently, the changing relationships between thyroid hormones must be considered when determining acceptable ranges (15). Systems with a hierarchical organization such as the HPT axis, tend to produce higher complexity, sensitivity, and variability at the higher levels and greater simplicity, stability, and constancy at the lower levels (13). Several complex non-linear relationships between system components may be required to obtain a simple output, namely a homeostatic state for a particular variable. Top-down or bottom-up regulation cannot be achieved simply through feedback and feedforward loops at the next upstream or downstream level of regulation. Rather it involves layers of feedback at two levels above or below them (13). In the case of the HPT axis the negative feedback from thyroid (level 3) goes up to both pituitary (level 2) and hypothalamus (level 1), whilst the positive feedforward from hypothalamus (level 1) goes down to both pituitary (level 2) and thyroid (level 3) (**Figure 1**). The integration of top-down and bottom-up regulation in time and space may explain typical ongoing stress-related phenomena. These can occur in non-thyroidal disease, including stress accumulation in form of allostatic load, a history of memory effects, and degeneration over time (13, 17, 20, 21). The classical HPT model regulation (22) describes the pituitary control of the production of the thyroid hormone FT4. Crucially, however, it does not consider feedback of thyroid hormones to the hypothalamus and a summation effect of neuronal brain afferences and other humoral brain signals impacting on TRH, either directly on a neuron or indirectly through neighboring tancytes (15, 17). It also does not go beyond thyroid hormone supply, as a means to integrate the utilization of thyroid hormones closer to the metabolic needs of the various tissues in the body (15, 17).

The proposed network of the HPT axis regulation is shown in **Figure 1**. We consider the mechanisms of defending FT3 homeostasis and possible adaptations. T3 is secreted by the human thyroid in a lesser quantity than T4. The critical importance of this apparently minor contribution of thyroidal T3 supply as a key element of control in the HPT axis regulation has hitherto been overlooked. T3 is less strongly bound than T4 to the transport proteins allowing T3 in its free form (FT3) to exert higher bioactivity (23). T3 is additionally produced in larger amounts by tissues other than thyroid, which can enzymatically deiodinate and activate the precursor T4 to T3 (24). Importantly, within the thyroid, TSH regulates both T4 production, T4 to T3 conversion, and direct synthesis of T3 (25). These mechanisms of regulating FT3 are essential for FT3 homeostasis and for systemic adaptations (15, 17), and are investigated in this study.

## Materials and Methods

### Mathematical Methods

The proof-of-concept minimal mathematical model reported here is in the form of 4 nonlinear, parameterized, first-order ordinary differential equations (ODEs) that account for the interactions of the 4 hormones TRH, TSH, FT4 and FT3 in the HPT axis regulation (**Figure 1**). A stable equilibrium solution of this system accounts for a physiological state, namely under normal conditions the euthyroid state. To examine mechanisms either critical for FT3 homeostasis or allowing to adapt the homeostatic level of FT3, 12 different scenarios are investigated by modeling 12 systems (subsystems), which account for different ways the system components could possibly interact within the network of the HPT axis. A modern method for both quantitative and qualitative investigation of such systems is numerical bifurcation analysis, which is based on the continuation of solutions to well-defined operator equations. The MATLAB-based (MATLAB, MathWorks, Natick, MA, USA) numerical bifurcation software Cl_MatContL (26, 27) is used in the present investigations. The equilibrium solutions are continued with respect to suitable input parameters, in our case, in particular, the FT4 production rate parameter k32, to investigate the influence on the FT3 homeostasis. The analytical solutions are produced by the MATLAB Symbolic Toolbox. For convenience of the reader, after completing the investigations, we have ported the ODE systems to the R programming language (28) with the added packages FME (29), deSolve (30) and rootSolve (30). R scripts are provided in **Supplementary Material** (doi: 10.5281/zenodo.6469235). We verified that the R results are of satisfactory quality to produce the required figures.

### Validation

Challenges to FT3 homeostasis investigated in these systems translate to specific clinical situations, such as the onset of autoimmune thyroiditis (31) and the non-thyroid illness syndrome (17). This allows a comparison with a previously published physiologically-based model (32) by our group and validation against clinical data (31, 33) (**Supplementary Materials**). No patient data were collected for this study. Deidentified data from published studies (31, 33) are available (**Supplementary Materials**). Across the different scales and units of the different models and data, outcomes had to be normalized, mean centered, and expressed as a percentage change. Statistical curve fitting is not appropriate for a proof-of-concept model, and regression lines shown in some figures are indicative only (see **Supplementary Material** for details).

## Results

### A Minimal Model of HPT Regulation

The regulatory HPT network (**Figure 1**) includes three endocrine organs, namely, the hypothalamus, pituitary, and thyroid gland, and four hormones, namely TRH, TSH, FT4, and FT3. We translate the physiological understanding (15) of the HPT axis regulation into a mathematical model [system (sys. 1)], as follows. Regulatory interactions consist of positive feedforward of TRH influence onto TSH, TSH influence onto FT4 and FT3, and negative feedback loops of FT4 and FT3 influences onto TSH and TRH (**Figure 1**). In theory, there are several ways in which the interplay between the system components can be described (15, 17). We therefore examine the influences of these components and criticality of different mechanisms for FT3 homeostasis in different subsystems, systems (sys. 2) - (sys. 12). For each of the systems (sys. 1) to (sys. 12) below we provide the analytical solution for FT3. Since the general (with all the parameters being free) analytical solutions are complicated, general solutions are only provided to demonstrate that FT3 does not depend on k32 for any values of all other parameters. In cases where we demonstrate dependence of FT3 on k32, we provide numerical examples with k32 and a3 being the only free parameters.

The regulatory system of the HPT axis is modelled mathematically by the system (sys. 1), below, of 4 coupled, first-order, parametrized, non-linear ordinary differential equations (ODEs). The system has 4 activating loops of the upstream hormones on downstream hormones, namely that of TRH on TSH, TSH on FT4, FT4 on FT3, and TSH on FT3 (**Figure 1**). It also has 4 repressing loops of downstream hormones on the upstream ones, namely of FT4 on TSH, FT3 on TSH, FT4 on TRH, and FT3 on TRH (**Figure 1**).


(eq. 1.1) $$\frac{d\left[TRH\right]}{dt}=\frac{s_{1}}{k_{134}\cdot \left[FT_{4}\right]\cdot \left[FT_{3}\right]}-a_{1}\cdot \left[TRH\right]$$

Equation (eq. 1.1) accounts for the rate of change of TRH. The first term on the right side of the equation represents the production rate, where s1 is an input signal from higher brain afferences, k134 is the proportionality constant of negative feedback of FT4 and FT3 onto TRH (upstream, repressing). We assume the AND gate relationship (34), as FT4 is converted to FT3, and it augments the activity of the latter (15). a1 is the elimination rate constant.


(eq. 1.2) $$\frac{d\left[TSH\right]}{dt}=k_{21}\cdot \left[TRH\right]+\frac{c_{2}}{k_{234}\cdot \left[FT_{4}\right]\cdot \left[FT_{3}\right])}-a_{2}\cdot \left[TSH\right]$$

Equation (eq. 1.2) accounts for the rate of change of TSH. The first term on the right is the production rate, where k21 is the proportionality constant of positive feedforward of TRH onto TSH (downstream, activating). The second term is the production rate due to negative feedback of FT4 and FT3 onto TSH (upstream, repressing), where c2 is the secretory capacity constant, and k234 is the proportionality constant. We assume the AND gate relationship (34), as FT4 is converted to FT3 and augments its activity (15). a2 is the elimination rate constant. Strong inhibition of both TRH at the hypothalamus in (eq 1.1) and TSH at the pituitary in (eq. 1.2) by FT3 and FT4 is a physiologically justifiable assumption in the euthyroid state (15), and FT3 is much higher than its Michaelis-Menten constant (32): FT3≫k; therefore 1/FT3 is a reasonable approximation of the Michaelis-Menten term 1/(k+FT3). The same is true for 1/FT4.


(eq. 1.3) $$\frac{d\left[FT_{4}\right]}{dt}=k_{32}\cdot \left[TSH\right]-a_{3}\cdot \left[FT_{4}\right]$$

Equation (1.3) accounts for the rate of change of FT4. The first term on the right is the production rate due to positive feedforward of TSH onto FT4 (downstream, activating), where k32 is the proportionality constant, and a3 is the elimination rate constant.


(eq. 1.4) $$\frac{d\left[FT_{3}\right]}{dt}=k_{423}\cdot \left[TSH\right]\cdot \left[FT_{4}\right]-a_{4}\cdot \left[FT_{3}\right]$$

Equation (1.4) accounts for the rate of change of FT3. The first term on the right is the production rate due to positive feedforward of TSH and FT4 onto FT3 (downstream, activating), where k423 is the proportionality constant, and a4 is the elimination rate constant.

System (sys. 1) has a unique equilibrium analytical solution with


(eq. 1.5) $$\left[FT_{3}\right]=\sqrt{\frac{k_{423}\cdot \left(a_{1}\cdot c_{2}\cdot k_{134}\;+\;s1\cdot \;k_{21}\cdot k_{234}\right)}{a_{1}\cdot a_{2}\cdot a_{4}\cdot k_{134}\cdot k_{234}}}$$

For solutions to other variables see **Supplementary Material**.

Equation (eq. 1.5) shows that the FT3 level is independent of the FT4 production rate constant k32 and the FT4 elimination constant a3. Hence, the FT3 homeostasis is preserved and not affected by changes in the FT4 levels. We call it “perfect homeostasis”.

This fact implies that the availability of FT3 is independent of a shortage in FT4 supply. Such a shortage occurs, for instance, at the onset of hypothyroidism. Conversely, if for instance an excess in FT4 supply occurs, at the onset of hyperthyroidism, FT3 homeostasis is also preserved according to equation (eq. 1.5).

**Figure 2:**
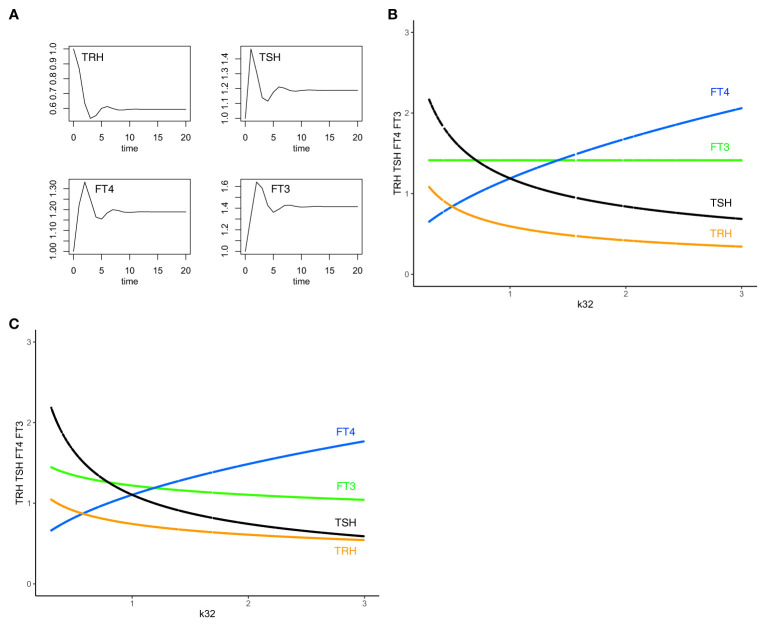
The minimal HPT model and mechanisms to preserve FT3 homeostasis. **(A)** The time-dependent solution of system (sys. 1) approaches stable equilibrium for all four hormones. **(B)** In system (sys. 1), homeostasis of FT3 (green line) is preserved as k32 (FT4 production rate constant) varies. This mechanism renders FT3 concentration insensitive to variations in the amount of its precursor FT4 (blue curve) and the levels of TSH (black curve) and TRH (orange curve) (“perfect FT3 homeostasis”). **(C)** In contrast, the mechanisms of (sys.1 mod) do not preserve FT3 homeostasis. Color code of hormones: TRH orange, TSH black, FT4 blue, FT3 green.

This equilibrium solution is found to be stable (all eigenvalues of the Jacobian matrix have negative real parts), as k32 varies from 0.3 to 3, with all other parameters fixed at 1 to demonstrate the principle. The time-dependent solution of system (sys. 1) approaches stable equilibrium (**Figure 2A**) and FT3 homeostasis is preserved in system (sys. 1), as k32 varies (**Figure 2B**).

From the physiological perspective, our minimal mathematical model accounts for mechanisms of the preservation of the FT3 homeostasis through a regulatory network of four interacting hormones (**Figure 1**). Such a mechanism is uniquely able to protect the biologically active hormone FT3, rendering it especially insensitive to variations in the amount of its precursor FT4 (**Figure 2B**).

In equation (eq. 1.2), we combined the two terms for the production rates of TSH, the positive feedforward by TRH and the negative feedback by FT4 and FT3, additively, which assumes they are largely independent. Physiologically, TRH acts at the pituitary through a transmembrane G protein-coupled receptor and several transcription factors (POU1F1, GATA2, CREB) to upregulate the TSH beta subunit gene and increase TSH production, and FT4 and FT3 act through an intracellular nuclear thyroid hormone receptor to downregulate the TSH beta subunit gene and repress TSH production (35). The different pathways suggest a parallel design with an additive coupling of their protein gene product TSH, as opposed to a serial design of dependent pathways with multiplicative coupling (35, 36). We are, however, not aware of any conclusive experimental results regarding how the positive and negative feedback loops are interlinked. Other mathematical models including our own models of the HPT axis (32, 37–39) phenomenologically approximated the nonlinear interaction with multiplicative terms for the two rate constants in the form of S-systems (40), particularly in pathological thyroid states where saturation effects, cooperativity and supra-additive interactions may be expected to occur.

For comparison, we also model the multiplicative combination of the positive and negative TSH production rate terms in system (sys. 1 mod), where equation (eq. 1.2) is replaced by (eq. 1.2 mod). All other equations are the same as in system (sys. 1).


(eq. 1.1 mod), $$\frac{d\left[TRH\right]}{dt}=\frac{s_{1}}{k_{134}\cdot \left[FT_{4}\right]\cdot \left[FT_{3}\right]}-a_{1}\cdot \left[TRH\right]$$

(eq. 1.2 mod), $$\frac{d\left[TSH\right]}{dt}=k_{21}\cdot \left[TRH\right]\cdot \frac{c_{2}}{k_{234}\cdot \left[FT_{4}\right]\cdot \left[FT_{3}\right])}-a_{2}\cdot \left[TSH\right]$$

(eq. 1.3 mod), $$\frac{d\left[FT_{4}\right]}{dt}=k_{32}\cdot \left[TSH\right]-a_{3}\cdot \left[FT_{4}\right]$$

(eq. 1.4 mod) $$\frac{d\left[FT_{3}\right]}{dt}=k_{423}\cdot \left[TSH\right]\cdot \left[FT_{4}\right]-a_{4}\cdot \left[FT_{3}\right]$$

We investigated the special case when k32 varies, a3 is free, and other parameters are fixed at 1.


(eq. 1.5 mod) $$\left[FT_{3}\right]={\left(\frac{a_{3}}{k_{32}}\right)}^{1/7}$$

System (sys. 1 mod), unlike system (sys. 1), does not preserve FT3 homeostasis, as k32 varies (**Figure 2C**), except for a physiologically unlikely special case of setting to a constant value of 1 the influence of TRH on TSH in (eq. 1.2 mod). Experimentally, models of combined additive or multiplicative responses are difficult to compare because results are often inconclusive (36). As a design principle (34), insensitivity to varying precursor concentrations, in our case of FT3 to FT4, can be achieved with system (sys. 1), but not system (sys. 1 mod).

### Analysis of System Components Critical for FT3 Homeostasis

To investigate the criticality of various components in the HPT regulation, system (sys. 1) is modified and extended, as will be described in the following subsystems (sys. 2) - (sys. 12). Because small changes in (sys. 1) can fundamentally alter the system behavior, we present those modifications as self-contained systems with analytical and graphical solutions included.

*System (sys. 2). Investigating the effects on FT3 homeostasis of setting to constant values the influences of thyroid hormones (FT4 and FT3) feedback on the hypothalamic hormone TRH*

There is little knowledge about the availability of thyroid hormones in the human brain, in particular the specific effects of FT4 and/or FT3 on TRH (17). Systems (sys. 2) - (sys. 7) investigate different mechanisms in this regard.

In system (sys. *2)*, FT4 and FT3 feedback levels onto TRH are possibly kept at constant values through physiological mechanisms (17), and we set those to 1. In this case, the TRH production rate is only changed by the input signal from higher brain afferences s1 and the elimination rate constant a1.

We replace equation (eq. 1.1) by equation (eq. 2.1). All other equations are the same as in system (sys. 1).


(eq. 2.1), $$\frac{d\left[TRH\right]}{dt}=s_{1}-a_{1}\cdot \left[TRH\right]$$

(eq. 2.2), $$\frac{d\left[TSH\right]}{dt}=k_{21}\cdot \left[TRH\right]+\frac{c_{2}}{k_{234}\cdot \left[FT_{4}\right]\cdot \left[FT_{3}\right])}-a_{2}\cdot \left[TSH\right]$$

(eq. 2.3), $$\frac{d\left[FT_{4}\right]}{dt}=k_{32}\cdot \left[TSH\right]-a_{3}\cdot \left[FT_{4}\right]$$

(eq. 2.4), $$\frac{d\left[FT_{3}\right]}{dt}=k_{423}\cdot \left[TSH\right]\cdot \left[FT_{4}\right]-a_{4}\cdot \left[FT_{3}\right]$$

**Figure 3:**
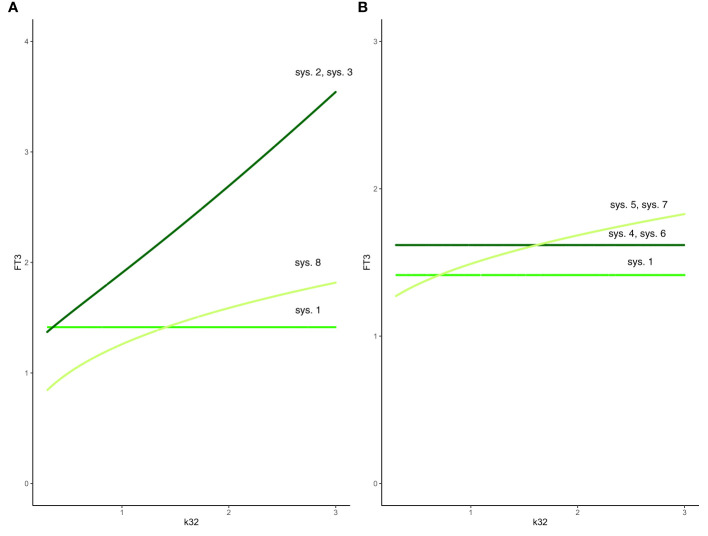
Effect of feedback and feedforward mechanisms on FT3 homeostasis. **(A)** Unlike in system (sys. 1) (green line), the absence of feedback by both FT4 and FT3 onto TRH in system (sys. 2) or TSH in system (sys. 3) results in a loss of FT3 homeostasis, as k32 (FT4 production rate constant) varies (coinciding dark-green curves). The removal of positive feedforward of TSH onto FT3 in system (sys. 8) also results in a loss of FT3 homeostasis, as k32 varies (olive-green curve). **(B)** In both cases that the influence of feedback by FT3 onto either TRH in system (sys. 4) or TSH in system (sys. 6) is constant, the level of FT3 homeostasis is shifted (dark-green curve), compared to system (sys. 1) (green curve), as k32 varies. In the other case that the influence of feedback by FT4 onto either TRH in system (sys. 5) or TSH in system (sys. 7) is constant system, FT3 homeostasis is lost (olive-green curve), as k32 varies.

System (sys. 2) has a unique equilibrium analytical solution. We investigated the special case when k32 varies from 0.3 to 3 and other parameters fixed at 1 (**Figure 3A**). In this case, with a3 free,


(eq. 2.5) $$\left[FT_{3}\right]=positive\;real\;root\;of(z^{4}-\frac{k_{32}}{a_{3}}\cdot z^{3}-2\cdot z^{2}+1)$$

Hence, the absence of feedback by both FT4 and FT3 onto TRH results in a loss of FT3 homeostasis (**Figure 3A**).

*System (sys. 3). Investigating the effects on FT3 homeostasis of setting to constant values the influence of thyroid hormones (FT4 and FT3) feedback on the pituitary hormone TSH*

We consider the physiologically unrealistic hypothetical case that the influence of feedback by both FT4 and FT3 on TSH remains constant, rendering its secretion constitutive.

We replace equation (eq. 1.2) by equation (eq. 3.2). All other equations are the same as in system (sys. 1).


(eq. 3.1), $$\frac{d\left[TRH\right]}{dt}=\frac{s_{1}}{k_{134}\cdot \left[FT_{4}\right]\cdot \left[FT_{3}\right]}-a_{1}\cdot \left[TRH\right]$$

(eq. 3.2), $$\frac{d\left[TSH\right]}{dt}=k_{21}\cdot \left[TRH\right]+\frac{c_{2}}{k_{234}\cdot \left[FT_{4}const\right]\cdot \left[FT_{3}const\right])}-a_{2}\cdot \left[TSH\right]$$

(eq. 3.3), $$\frac{d\left[FT_{4}\right]}{dt}=k_{32}\cdot \left[TSH\right]-a_{3}\cdot \left[FT_{4}\right]$$

(eq. 3.4), $$\frac{d\left[FT_{3}\right]}{dt}=k_{423}\cdot \left[TSH\right]\cdot \left[FT_{4}\right]-a_{4}\cdot \left[FT_{3}\right]$$

We investigated the special case when k32 varies from 0.3 to 3, and all other parameters fixed at 1 (**Figure 3A**). In this case,


(eq. 3.5) $$\left[FT_{3}\right]=positive\;real\;root\;of\;(z^{4}-\frac{k_{32}}{a_{3}\cdot FT_{3const}^{2}\cdot FT_{4const}^{2}}\cdot z^{3}-2\cdot z^{2}+1)$$

The absence of feedback by both thyroid hormones FT4 and FT3 onto TSH results in a loss of FT3 homeostasis (**Figure 3A**). Such a constitutive mechanism is not regarded as physiologically meaningful in the HPT axis regulation (15).

*System (sys. 4). Investigating the effect on FT3 homeostasis of setting to constant value (FT3_const) thyroid hormone FT3 feedback on the hypothalamic hormone TRH*

We investigate the case that the influence of feedback by FT3 is constant, while that of FT4 varies (17).

We replace equation (eq. 1.1) with equation (eq. 4.1). All other equations are the same as in system (sys. 1).


(eq. 4.1), $$\frac{d\left[TRH\right]}{dt}=\frac{s_{1}}{k_{13}\cdot \left[FT_{3}const\right]\cdot \left[FT_{4}\right]}-a_{1}\cdot \left[TRH\right]$$

(eq. 4.2), $$\frac{d\left[TSH\right]}{dt}=k_{21}\cdot \left[TRH\right]+\frac{c_{2}}{k_{234}\cdot \left[FT_{4}\right]\cdot \left[FT_{3}\right]}-a_{2}\cdot \left[TSH\right]$$

(eq. 4.3), $$\frac{d\left[FT_{4}\right]}{dt}=k_{32}\cdot \left[TSH\right]-a_{3}\cdot \left[FT_{4}\right]$$

(eq. 4.4), $$\frac{d\left[FT_{3}\right]}{dt}=k_{423}\cdot \left[TSH\right]\cdot \left[FT_{4}\right]-a_{4}\cdot \left[FT_{3}\right]$$

We investigated the special case when k32 varies from 0.3 to 3 with all other parameters fixed at 1 (**Figure 3B**).

In this case,


(eq. 4.5) $$\left[FT_{3}\right]=\frac{{\left(4\cdot FT_{3const}^{2}+1\right)}^{1/2}+1}{2\cdot FT_{3const}}$$

In system (sys. 4), as in system (sys. 1), FT3 does not depend on the FT4 production rate constant k32, and the FT4 elimination rate constant a3. Equation (eq. 4.5) shows that FT3 level depends on the amount (FT3_const) of constant FT3 feedback. For = FT3_const = 1, the constant (solution) FT3 level is 1.6180; for = FT3_const = 0.5, the constant (solution) FT3 level is 2.4142; for = FT3_const = 1.5, the constant (solution) FT3 level is 1.3874. In all cases, the FT3 homeostasis is preserved. There are physiological ways to vary this constant. Those are facilitated by the blood-brain barrier and the requirement for thyroid hormones FT4 and FT3 to be actively transported across the plasma membrane to enter the cell (15). Thyroid hormone transporters are further known to differ in their affinities for T4 and T3, and they are expressed in an organ-specific and species-specific manner (41). A rare disease, the Allan-Herndon-Dudley syndrome shows both mechanistic and phenotypic properties of this system (41).

*System (sys. 5). Investigating the effect on FT3 homeostasis of setting the level of thyroid hormone FT4 feedback on the hypothalamic hormone TRH to a constant*

We investigate the physiologically less likely case that the influence of feedback by FT4 is constant, in our case 1, while that of FT3 varies (15).

We replace equation (eq. 1.1) with equation (eq. 5.1). All other equations are the same as in system (sys. 1).


(eq. 5.1), $$\frac{d\left[TRH\right]}{dt}=\frac{s_{1}}{k_{14}\cdot \left[FT_{3}\right]}-a_{1}\cdot \left[TRH\right]$$

(eq. 5.2), $$\frac{d\left[TSH\right]}{dt}=k_{21}\cdot \left[TRH\right]+\frac{c_{2}}{k_{234}\cdot \left[FT_{4}\right]\cdot \left[FT_{3}\right]}-a_{2}\cdot \left[TSH\right]$$

(eq. 5.3), $$\frac{d\left[FT_{4}\right]}{dt}=k_{32}\cdot \left[TSH\right]-a_{3}\cdot \left[FT_{4}\right]$$

(eq. 5.4), $$\frac{d\left[FT_{3}\right]}{dt}=k_{423}\cdot \left[TSH\right]\cdot \left[FT_{4}\right]-a_{4}\cdot \left[FT_{3}\right]$$

FT3 can depend on k32. We investigated the special case when k32 varies from 0.3 to 3 with all other parameters fixed at 1 (**Figure 3B**).


(eq. 5.5) $$\left[FT_{3}\right]\;=\;positive\;real\;root\;of\;\left(a_{3}\cdot z^{4}-2\cdot a_{3}\cdot z^{2}-k_{32}\cdot z+a_{3}\right)$$

Such a system, with no negative feedback of FT4 onto TRH, does not preserve FT3 homeostasis, though the equilibrium solution is still stable when FT4 production rate constant k32 varies. In this case, the level of FT3 depends on k32 (**Figure 3B**). This is not regarded as a physiologically favorable mechanism of regulation in the HPT axis (15).

*System (sys. 6). Investigating the effect on the FT3 homeostasis of setting to constant values the influence of FT3 feedback on the pituitary hormone TSH*

We investigate the case that the influence of feedback by FT3 remains constant, while that of FT4 varies (15).

We replace equation (eq. 1.2) by equation (eq. 6.2). All other equations are the same as in system (sys. 1).


(eq. 6.1), $$\frac{d\left[TRH\right]}{dt}=\frac{s_{1}}{k_{134}\cdot \left[FT_{4}\right]\cdot \left[FT_{3}\right]}-a_{1}\cdot \left[TRH\right]$$

(eq. 6.2), $$\frac{d\left[TSH\right]}{dt}=k_{21}\cdot \left[TRH\right]+\frac{c_{2}}{k_{23}\cdot \left[FT_{3}const\right]\cdot \left[FT_{4}\right]}-a_{2}\cdot \left[TSH\right]$$

(eq. 6.3), $$\frac{d\left[FT_{4}\right]}{dt}=k_{32}\cdot \left[TSH\right]-a_{3}\cdot \left[FT_{4}\right]$$

(eq. 6.4), $$\frac{d\left[FT_{3}\right]}{dt}=k_{423}\cdot \left[TSH\right]\cdot \left[FT_{4}\right]-a_{4}\cdot \left[FT_{3}\right]$$

System (sys. 6) has a unique equilibrium analytical solution, where FT3 does not depend on k32 and a3, as in system (sys. 1). The FT3 component of the general solution is:


(eq. 6.5) $$\left[FT_{3}\right]\;=\;\frac{k_{423}^{1/2}\cdot {\left(4\cdot a_{2}\cdot a_{4}\cdot k_{21}\cdot s_{1}\cdot FT_{3const}^{2}\cdot k_{23}^{2}+a_{1}\cdot k_{134}\cdot k_{423}\cdot c_{2}^{2}\right)}^{1/2}+a_{1}^{1/2}\cdot c_{2}\cdot k_{134}^{1/2}\cdot k_{423}}{2\cdot FT_{3const}\cdot a_{1}^{1/2}\cdot a_{2}\cdot a_{4}\cdot k_{23}\cdot k_{134}^{1/2}}$$

We investigated the special case when k32 varies from 0.3 to 3 with all other parameters fixed at 1 (**Figure 3B**). Equation (eq. 6.5) shows that FT3 level depends on the amount (FT3_const) of constant FT3 feedback. For = FT3_const = 1, the FT3 level is 1.6180, for = FT3_const = 0.5, the FT3 level is 2.4142; for = FT3_const = 1.5, the FT3 level is 1.3874. In all cases, the FT3 homeostasis is preserved, and its level is shifted depending on the value of FT3_const. There are physiological ways to modify pituitary FT3 to adjust the level of FT3 homeostasis (42).

*System (sys. 7). Investigating the effect on the FT3 homeostasis of setting the level of thyroid hormone FT4 feedback on the pituitary hormone TSH to a constant*

We consider the physiologically unrealistic hypothetical case that the influence of feedback by FT4 is constant, in our case 1, while that of FT3 varies (15).

We replace equation (eq. 1.2) by equation (eq. 7.2). All other equations are the same as in system (sys. 1).


(eq. 7.1), $$\frac{d\left[TRH\right]}{dt}=\frac{s_{1}}{k_{134}\cdot \left[FT_{4}\right]\cdot \left[FT_{3}\right]}-a_{1}\cdot \left[TRH\right]$$

(eq. 7.2), $$\frac{d\left[TSH\right]}{dt}=k_{21}\cdot \left[TRH\right]+\frac{c_{2}}{k_{24}\cdot \left[FT_{3}\right]}-a_{2}\cdot \left[TSH\right]$$

(eq. 7.3), $$\frac{d\left[FT_{4}\right]}{dt}=k_{32}\cdot \left[TSH\right]-a_{3}\cdot \left[FT_{4}\right]$$

(eq. 7.4), $$\frac{d\left[FT_{3}\right]}{dt}=k_{423}\cdot \left[TSH\right]\cdot \left[FT_{4}\right]-a_{4}\cdot \left[FT_{3}\right]$$

System (sys. 7) has a unique equilibrium analytical solution. We investigated the special case when k32 varies from 0.3 to 3 with all other parameters fixed at 1 (**Figure 3B**). In this case,


(eq. 7.5) $$\left[FT_{3}\right]=positive\;real\;root\;of\;(z^{4}-2\cdot z^{2}-\frac{k_{32}}{a_{3}}\cdot z+1)$$

System (sys. 7), with no negative feedback of FT4 onto TSH, does not preserve FT3 homeostasis, as FT4 production rate constant k32 varies. In this case, the level of FT3 depends on k32 (**Figure 3B**). This is not regarded as a physiologically favorable mechanism of regulation in the HPT axis (15).

*System (sys. 8). Investigating the effect on FT3 homeostasis of removing the positive pituitary TSH feedforward on the thyroid hormone FT3*

We investigate the effect of a loss of TSH-feedforward on FT3, which is physiologically realistic in patients with thyroid failure (15).

We replace equation (eq. 2.4) by equation (eq. 8.4). All other equations are the same as in system (sys. 1).


(eq. 8.1), $$\frac{d\left[TRH\right]}{dt}=\frac{s_{1}}{k_{134}\cdot \left[FT_{4}\right]\cdot \left[FT_{3}\right]}-a_{1}\cdot \left[TRH\right]$$

(eq. 8.2), $$\frac{d\left[TSH\right]}{dt}=k_{21}\cdot \left[TRH\right]+\frac{c_{2}}{k_{234}\cdot \left[FT_{4}\right]\cdot \left[FT_{3}\right]}-a_{2}\cdot \left[TSH\right]$$

(eq. 8.3), $$\frac{d\left[FT_{4}\right]}{dt}=k_{32}\cdot \left[TSH\right]-a_{3}\cdot \left[FT_{4}\right]$$

(eq. 8.4), $$\frac{d\left[FT_{3}\right]}{dt}=k_{43}\cdot \left[FT_{4}\right]-a_{4}\cdot \left[FT_{3}\right]$$

We investigated the special case when k32 varies from 0.3 to 3 with all other parameters fixed at 1 (**Figure 3A**). In this case,


(eq. 8.5) $$\left[FT_{3}\right]={\left(\frac{2\cdot k_{32}}{a_{3}}\right)}^{1/3}$$

System (sys. 8), with no positive feedforward of TSH onto FT3, does not preserve FT3 homeostasis, though the equilibrium solution is still stable as FT4 production rate constant k32 varies (**Figure 3A**). Physiologically, a loss of the positive feedback of TSH onto FT3 occurs in patients without sufficient residual thyroid tissue, for instance after thyroidectomy, and reduces their T4 to T3 converting capabilities (32).

In summary, we investigated 8 different mechanisms in the HPT axis regulation to preserve FT3 homeostasis in the face of variations/changes of the FT4 production rate. Only the mechanisms in the system (sys. 1), system (sys. 4), and system (sys. 6) support the preservation of FT3 homeostasis in the HPT axis regulation (**Figure 3**).

### Allowing Adaptation

A system designed for perfect homeostasis, while optimally protecting its target, does not provide a mechanism for discerning a possible need for an adaptation, or actually producing an adaptation, i.e. shifting due to circumstances to a more favorable homeostatic state. Accordingly, an adaptive system response, termed allostasis (8), requires additional mechanisms that may provide the desired adaptivity, flexible and variable in time and space (17). A particular aspect of adaptation within an organism relates to the diversity of the energetic and metabolic needs amongst the various organs of the body in the hierarchical organization of the hypothalamic-pituitary-thyroid axis (43). Such an adaptation can be facilitated by a certain degree of local control, involving the decentralized process of enzymatic peripheral T4 to T3 conversion, resulting in extra-thyroidal FT3 production, taking place directly at the level of each organ (43). T4 to T3 conversion is regulated by three different types of deiodinases expressed by many tissues (43). It depends on the availability of sufficient amounts of the precursor T4, the latter, in turn, being dependent on appropriate TSH stimulation (15). Thereby, this process is interconnected with the regulatory system of the HPT axis. The summation effect of extra-thyroidal FT3 production feeds back to the pituitary and hypothalamus, providing bottom-up information on the hormone-dependent energetic state of the organs. To raise circulating FT4 and FT3 levels sufficiently to achieve a healthy level, top-down regulation by TRH and TSH is required, its absence causing a hypothyroid state (low FT4 and low FT3) (15). The amount of non-thyroidal peripheral FT3 production varies by species and condition. FT3 efflux from tissues has been estimated to be larger in humans, compared to rodents, contributing up to 80% to the circulating T3 pool in humans (24).

The minimum model is further extended to account for a combination of mechanisms of preserving homeostasis of the HPT axis regulation and allowing adaptation.

*System (sys. 9). Investigating the effect of peripheral FT3 production on FT3 homeostasis*

**Figure 4:**
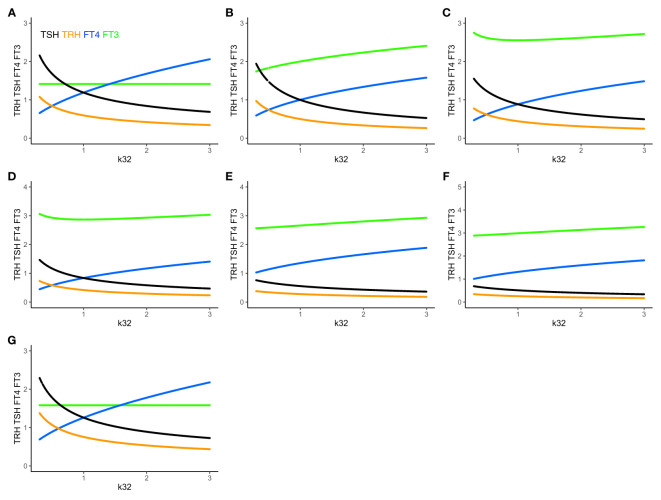
The extended HPT model and mechanisms allowing for adaptation. Unlike **(A)** system (sys. 1), **(B)** system (sys. 9), which accounts for additional TSH-independent peripheral (non-thyroidal) FT3 production by various organs, does not preserve FT3 homeostasis. The level of FT3 (green curve) is adapted, as k32 varies, depending on the value of k43 (here k43 = 1). **(C)** System (sys. 10) additionally accounts for FT4-independent, TSH-dependent thyroidal FT3 production. This mechanism shifts FT3 homeostasis, depending on the values of k32 and k42 (here k42 = 1). It especially protects the level of FT3 against a shortage in the availability of the precursor FT4, when k32 decreases <1. **(D)** System (sys. 11) introduces a T3 drug, modelled, in the presence of an intact thyroid gland, as a constant external production rate of FT3 (drug_3 =_ 0.5) **(E)** introduces in system (sys. 11) a T4 drug as a constant external production rate of FT4 (drug_4 =_ 0.8), and **F**) a combination of both drugs (drug_4 =_ 0.8, drug_3 =_ 0.5). The T3 drug is more protective of the FT3 level than the FT4 dug or the combination. **(G)** In system (sys. 12), which accounts for variation in the TRH production rate constant due to seasonal change, FT3 homeostasis is shifted to a new level depending on the value of ptTSH (here ptTSH=1.5). Color code of hormones: TRH orange, TSH black, FT4 blue, FT3 green.

The minimal model (**Figure 4A**) is extended to account for non-thyroidal peripheral FT3 production by adding the term k43*[FT4], which physiologically depends on FT4, but not on TSH (15).

We replace equation (eq. 1.4) by equation (eq. 9.4). All other equations are the same as in system (sys. 1).


(eq. 9.1), $$\frac{d\left[TRH\right]}{dt}=\frac{s_{1}}{k_{134}\cdot \left[FT_{4}\right]\cdot \left[FT_{3}\right]}-a_{1}\cdot \left[TRH\right]$$

(eq. 9.2), $$\frac{d\left[TSH\right]}{dt}=k_{21}\cdot \left[TRH\right]+\frac{c_{2}}{k_{234}\cdot \left[FT_{4}\right]\cdot \left[FT_{3}\right]}-a_{2}\cdot \left[TSH\right]$$

(eq. 9.3), $$\frac{d\left[FT_{4}\right]}{dt}=k_{32}\cdot \left[TSH\right]-a_{3}\cdot \left[FT_{4}\right]$$

(eq. 9.4), $$\frac{d\left[FT_{3}\right]}{dt}=k_{423}\cdot \left[TSH\right]\cdot \left[FT_{4}\right]+k_{43}\cdot \left[FT_{4}\right]-a_{4}\cdot \left[FT_{3}\right]$$

We investigated the special case when k32 varies from 0.3 to 3 with other parameters fixed at 1 (**Figure 4B**). In this case, with k32 = 1,


(eq. 9.5) $$\left[FT_{3}\right]=positive\;real\;root\;of\;(a_{3}\cdot z^{4}-4\cdot a_{3}\cdot z^{2}-2\cdot z+4\cdot a_{3})$$

Adding the term k43*[FT4] in (eq. 9.4) increases the FT3 production rate, and the level of FT3 goes up, which breaks FT3 homeostasis (**Figures 4A, B**). There are known physiological mechanisms (15) to effectively vary k32 and k43. We hypothesize, that the organism can use these mechanisms to adapt the level of FT3 by continuously varying k43 in peripheral organs, such as the liver, and/or k32 in the thyroid (to change the level of FT4 which depends on k32 in (eq. 9.3)), until FT3 reaches the desired level, which may be different in different situations (17).

We investigated two special cases: when a) k43 = 1, and k32 varies from 0.3 to 3 (**Figure 4B**) or b) k43 varies from 0.3 to 3 with all other parameters fixed at 1. In both cases, while the FT3 level increases, the equilibrium solution is still stable, and FT3 increases, as k43 or k32 increases.

Physiologically, this allows the peripheral organs to adapt the level of FT3 if their needs change. k43*[FT4] can be low, if FT4 concentration is low, as in hypothyroidism, or if k43 is small, as in severe non-thyroidal illness. In both cases, this means the extra-thyroidal FT3 production rate is insufficient and the system adapts to shift FT3 homeostasis.

*System (sys. 10). Investigating the effect of de novo FT3 secretion on FT3 homeostasis*

The human thyroid gland produces a large amount of T4, and a small amount of T3 (24). System (sys. 9) is further extended to account for the *de novo* synthesis of FT3 in the thyroid gland, by adding the term k42*[TSH] in equation (eq. 9.4), which is physiologically independent of FT4, but depends on TSH (44).

We replace equation (eq. 9.4) by equation (eq. 10.4). All other equations are the same as in system (eq. 9).


(eq. 10.1), $$\frac{d\left[TRH\right]}{dt}=\frac{s_{1}}{k_{134}\cdot \left[FT_{4}\right]\cdot \left[FT_{3}\right]}-a_{1}\cdot \left[TRH\right]$$

(eq. 10.2), $$\frac{d\left[TSH\right]}{dt}=k_{21}\cdot \left[TRH\right]+\frac{c_{2}}{k_{234}\cdot \left[FT_{4}\right]\cdot \left[FT_{3}\right]}-a_{2}\cdot \left[TSH\right]$$

(eq. 10.3), $$\frac{d\left[FT_{4}\right]}{dt}=k_{32}\cdot \left[TSH\right]-a_{3}\cdot \left[FT_{4}\right]$$

(eq. 10.4) $$\frac{d\left[FT_{3}\right]}{dt}=k_{423}\cdot \left[TSH\right]\cdot \left[FT_{4}\right]+k_{43}\cdot \left[FT_{4}\right]+k_{42}\cdot \left[TSH\right]-a_{4}\cdot \left[FT_{3}\right]$$

We investigated the special case when k32 varies from 0.3 to 3 with other parameters fixed at 1 (**Figure 4C**). With k32 = 1,


(eq. 10.5) $$\left[FT_{3}\right]=positive\;real\;root\;of\;(a_{3}\cdot z^{4}-4\cdot a_{3}\cdot z^{2}-2\cdot a_{3}^{2}\cdot z-4\cdot a_{3}\cdot z-2\cdot z+4\cdot a_{3})$$

Adding the term k42*[TSH] in (eq. 10.4) increases the FT3 production rate, and the level of FT3 goes up, which breaks FT3 homeostasis. There are known physiological mechanisms (15) to effectively vary k32 and k42. We hypothesize, that the organism can use these mechanisms to adapt the level of FT3 by varying k42 and/or k32 (to change the level of FT4, which depends on k32 in (eq. 10.3), and, in turn, affects the level of TSH in (eq. 10.2), which depends on the FT4 feedback in (eq. 10.2)) in the thyroid, until FT3 reaches the desired level, which may be different in different situations (17).

We investigated two special cases: when a) k42 = 1, and k32 varies from 0.3 to 3 (**Figure 4C**) and b) k42 = 2, and k32 varies from 0.3 to 3, with all other parameters fixed at 1. The equilibrium solution is still stable, and FT3 increases, as k32 moves away from 1, going up both from k32<1 and k32>1. In both cases, the FT3 level increases asymmetrically, i.e. more sharply with k32<1, compared to k32>1. k42 = 2 produces a sharp FT3 increase for k32<1, but a relatively flat response for k32>1.

Physiologically, such mechanisms allow the thyroid to shift the homeostasis of FT3, if needed. k42*[TSH] can be increased, if TSH or/and k42 is large. In both cases, this means that the FT3 production rate contributed by intrathyroidal and peripheral FT4-dependent conversion is insufficient, and, the system adapts to shift FT3 homeostasis by increasing the fraction of the contribution of the TSH-dependent FT3 production. Physiologically, this is regarded as a compensatory mechanism, which has been shown to protect the level of FT3 at the onset of hypothyroidism in patients with autoimmune thyroiditis facing a shortage of FT4 production due to a progressive destruction of thyroid tissue (31, 32).

System (sys. 11). Investigating the effects of external T4 and/or T3 administration on FT3 homeostasis

As a control experiment for modelling, we introduce a T4 drug (drug4) and a T3 drug (drug3) as constant external production rates of FT3 and FT4. This is accomplished by replacing (eq. 10.3) by (eq. 11.3), and (eq. 10.4) by (eq. 11.4). All other equations are the same as in system (10).


(eq. 11.1), $$\frac{d\left[TRH\right]}{dt}=\frac{s_{1}}{k_{134}\cdot \left[FT_{4}\right]\cdot \left[FT_{3}\right]}-a_{1}\cdot \left[TRH\right]$$

(eq. 11.2), $$\frac{d\left[TSH\right]}{dt}=k_{21}\cdot \left[TRH\right]+\frac{c_{2}}{k_{234}\cdot \left[FT_{4}\right]\cdot \left[FT_{3}\right]}-a_{2}\cdot \left[TSH\right]$$

(eq. 11.3), $$\frac{d\left[FT_{4}\right]}{dt}=k_{32}\cdot \left[TSH\right]+drug_{4}-a_{3}\cdot \left[FT_{4}\right]$$

(eq. 11.4), $$\frac{d\left[FT_{3}\right]}{dt}=k_{423}\cdot \left[TSH\right]\cdot \left[FT_{4}\right]+k_{43}\cdot \left[FT_{4}\right]+k_{42}\cdot \left[TSH\right]+drug_{3}-a_{4}\cdot \left[FT_{3}\right]$$

We investigated several special cases, as k32 varies from 0.3 to 3 with other parameters fixed at 1, and display the graphical solutions (**Figures 4D–F**).

Adding a T3 drug (drug3 = 0.5, **Figure 4D**) protects the level of FT3, whereas adding a T4 drug (drug4 = 0.8, **Figure 4E**) or a T3 and T4 drug (drug4 = 0.8 plus drug3 = 0.3, **Figure 4F**) increases the level of FT4 and/or FT3, but is less FT3-protective, as k32 decreases. This accounts for the additional effect of a T3- and/or T4-containing drug at equilibrium in the presence of a thyroid gland, which does not include the clinical situation of a patient without a thyroid gland on thyroid hormone replacement, as modelled elsewhere (32).

We have investigated the effects of different sources of FT3 (sys. 1, sys. 9, sys. 10, sys. 11), involving different physiological mechanisms, on the preservation or adaptation of FT3 homeostasis. The different mechanisms provide the flexibility for the body to choose between mechanisms to preserve FT3 homeostasis or adapt FT3 to a different level, whichever is more appropriate, depending on its needs in different physiological situations. We note that there is a range sensitivity of the mechanism(s) and adaptivity of the contributions of each relevant mechanism (32). The equilibria of FT3, FT4, TSH, and TRH, expressed by the HPT axis, vary depending on the specific conditions. Hence, their appropriateness can no longer be simply established by reference to the equilibrium state which is expressed in thyroid health *via* established normative reference values (45). The dependence of the equilibria on different physiological situations, revealed in the present model, is supported by clinical studies and has been described by the concept of relational stability (45). For example, a slightly elevated TSH could be a beneficial adaptation in a patient with subclinical hypothyroidism, rather than a clinical disease (45).

### Adaptation to Seasonality

Adaptation to seasonality in the system response involves top-down regulation, as has been documented in various animal species, particularly birds (46). A postulated mechanism involves tancytes, which respond to ptTSH (47, 48) and are also known to regulate hypothalamic TRH (49, 50). ptTSH is a variant form of TSH produced in the pars tibialis of the pituitary, different from the TSH of the pituitary pars tuberalis, devoid of thyroid-stimulating activity, and suppressed by melatonin (47, 48). We model such variations by a parameter ptTSH included in the TRH production rate in system (sys. 12). This accounts, in a simplified manner, for the aggregation of the underlying physiological mechanisms.

*System (sys. 12). Investigating the effect of varying the TRH production rate*

We replace equation (eq. 1.1) by (eq. 12.1). All other equations are the same as in system (sys. 1).


(eq. 12.1), $$\frac{d\left[TRH\right]}{dt}=\frac{ptTSH\cdot s_{1}}{k_{134}\cdot \left[FT_{4}\right]\cdot \left[FT_{3}\right]}-a_{1}\cdot \left[TRH\right]$$

(eq. 12.2), $$\frac{d\left[TSH\right]}{dt}=k_{21}\cdot \left[TRH\right]+\frac{c_{2}}{k_{234}\cdot \left[FT_{4}\right]\cdot \left[FT_{3}\right]}-a_{2}\cdot \left[TSH\right]$$

(eq. 12.3), $$\frac{d\left[FT_{4}\right]}{dt}=k_{32}\cdot \left[TSH\right]-a_{3}\cdot \left[FT_{4}\right]$$

(eq. 12.4), $$\frac{d\left[FT_{3}\right]}{dt}=k_{423}\cdot \left[TSH\right]\cdot \left[FT_{4}\right]-a_{4}\cdot \left[FT_{3}\right]$$

System (sys. 12) has a unique equilibrium analytical solution, which follows from system (sys. 1),


(eq. 12.5) $$\left[FT_{3}\right]\;=\;\sqrt{\frac{k_{423}\cdot \left(a_{1}\cdot c_{2}\cdot k_{134}\;+ptTSH\;\cdot s1\cdot \;k_{21}\cdot k_{234}\right)}{a_{1}\cdot a_{2}\cdot a_{4}\cdot k_{134}\cdot k_{234}}}$$

ptTSH is a parameter, which varies by season, and modulates s1 in (eq. 12.1). In system (sys. 12), FT3 homeostasis is preserved and shifted to a different level depending on the value of ptTSH in (eq. 12.1). We investigated three special cases: when a) ptTSH = 1, b) ptTSH = 0.5, and c) ptTSH =1.5, as k32 varies from 0.3 to 3 with all other parameters fixed at 1. For ptTSH = 1, the constant (solution) FT3 level is 1.4142; for ptTSH = 0.5, the constant (solution) FT3 level is 1.2247; for = ptTSH = 1.5, the constant (solution) FT3 level is 1.5811 (**Figure 4G**).

This means that naturally occurring variations in the top-level input, which influence the TRH production rate constant, can be used to sensitively and precisely adapt the level of FT3 homeostasis.

### Physiological Considerations of the System Regulation Versus a Local Regulation

**Figure 5:**
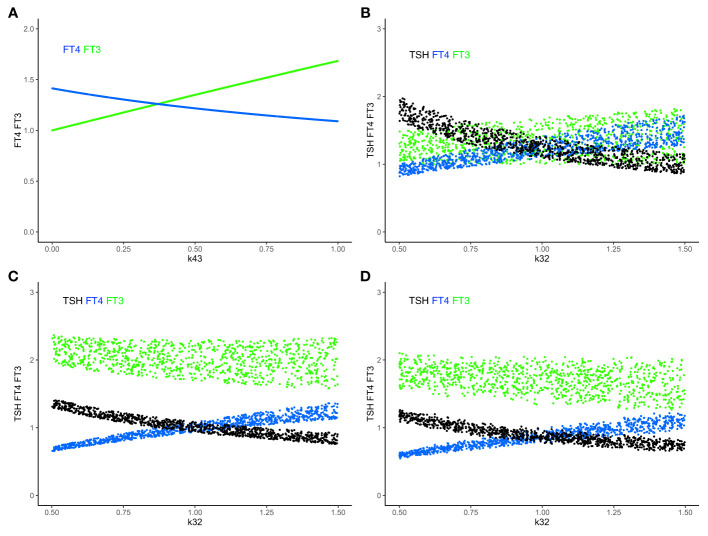
Combining mechanisms of FT3 homeostasis and FT3 adaptation. **(A)** In system (sys. 9), with k32 = 1, k423 = 0.5, k42 = 0 and other parameters =1, increasing k43 (the FT3 production rate constant of peripheral organs) increases the level of FT3 (green curve), while the level of FT4 decreases (blue curve). FT3 and FT4 move in opposite directions. This mechanism disjoins the levels of FT3 and FT4. **(B)** The level of FT3 is protected by this mechanism in system (sys. 9) within a certain range, as both k43 = 0-1 (as in A) and k32 (FT4 production rate constant) vary simultaneously. **(C)** In system (sys. 10), the TSH feedforward on FT3 is switched on (k42 = 1). This mechanism increases the level of FT3 more strongly, as k32 decreases, compared to B (sys. 9, k42 = 0). **(D)** In system (sys. 10), decreasing the range of s1 (TRH production rate constant) (here s1 = 0.5 to 0.1, with other parameters as in **(C)** shifts and reduces the range of FT3 levels, compared to C. Color code of hormones: TSH black, FT4 blue, FT3 green.

In systems (sys. 1) - (sys. 12), we have investigated regulatory principles of homeostasis. In the human organism, preservation of homeostasis (in our case of FT3) controlled by the centralized system (in our case at the hypothalamus and pituitary) can be combined with mechanisms of adaptation at lower levels of the hierarchy in the organization (in our case non-thyroidal organs producing their own FT3). In particular, peripheral FT3 production, accounted for in (eq. 9.4), provides individual non-thyroidal organs with a certain level of regulatory autonomy to produce more or less FT3 to meet their different metabolic needs. This may have proven advantageous in the evolution of HPT axis regulation (51). FT3 released from organs other than the thyroid, feeds back onto TSH, which, in turn, decreases the production of its precursor FT4. When increasing the amount of peripheral non-thyroidal FT3 production, FT3 and FT4 levels move in opposite directions (**Figure 5A**). When additionally varying thyroidal FT4 production rate k32 (eq. 9.3), the resulting ranges of FT3 and FT4 as functions of k32 are shown in **Figure 5B**. The range of the homeostatic levels of FT3 can be adapted, as k32 varies, by switching on and simultaneously varying the feedforward of TSH on FT3 (eq. 10.4) (**Figure 5C**). The range of FT3 homeostasis can be further adapted by varying the central influences on the TRH production rate constant s1 (eq. 10.1) (**Figure 5D**).

A life-saving potential of the TSH-dependent rescue mechanism to raise the level of FT3 independently of FT4 (k42*TSH, (eq. 10.4)), if needed, has been experimentally confirmed in genetically modified animals with intact thyroid and completely deficient deiodinase functionalities (52). Similar considerations apply to iodine deficiency, which is accompanied by up-regulated deiodinase activity (53).

Taken together, our findings support the concept that the system regulating the HPT axis has suitable mechanisms to assess and respond to the efficiency challenge of combining FT3 produced by different organs in the organism, enabling it to correct its targets for the supply of the body with thyroid hormones.

### Validation of System Principles

**Figure 6:**
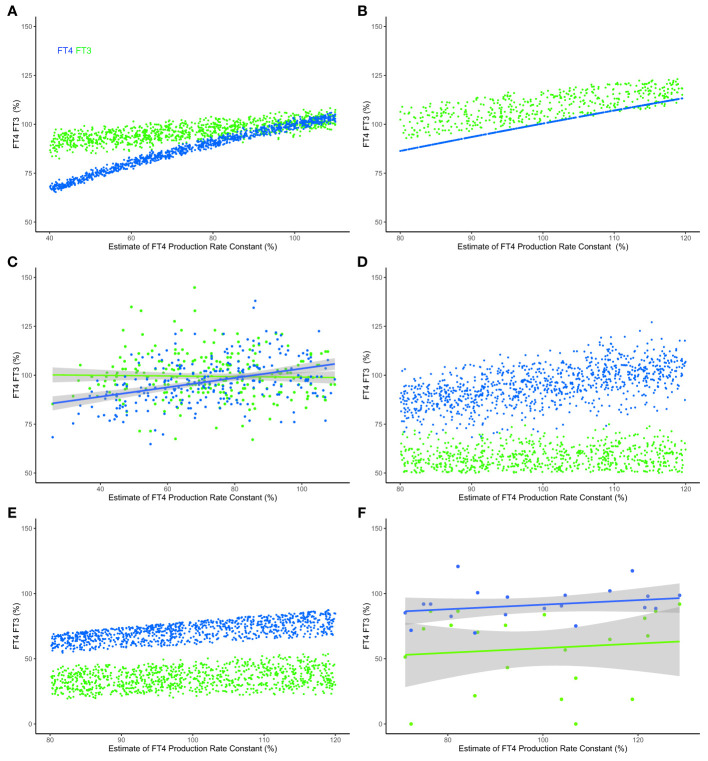
Modeled and clinical outcomes of FT3 homeostasis and FT3 adaptation. **(A)** FT4 levels (blue curve) decline, but the mechanisms in system (sys. 10) protect FT3 levels (green curve), keeping them in a range close to the original level, as the percentage of the estimate of FT4 production rate constant (k32 = 0.4-1.1) decreases - as typical at the onset of hypothyroidism in patients suffering from autoimmune thyroiditis and progressive thyroid destruction. Other parameters are chosen within ranges, k423 = 0.2-0.3, k43 = 0.7-0.8 and k42 = 0.1-0.2, to approximate the relative contributions to FT3 production according to (32). **(B)** The predictions of a physiologically-based molecular model (32) are comparable with those of the minimal model, as the percentage estimate of FT4 production rate constant (termed v0 in this model) decreases. **(C)** The FT3 and FT4 solutions predicted by the models were clinically observed in a sample of patients with thyroid autoimmune disease (31). In the clinical data, a percentage estimate of FT4 production rate constant is obtained by normalizing the maximum secretory capacity of the thyroid gland (termed GT) (31). **(D)** The levels of FT3 are low, as the percentage estimate of FT4 production rate constant (k32 = 0.8-1.2) varies, and multiple other rate constants, k423 = 0.2-0.3, k43 = 01-0.4, k42 = 0.1-0.2, s1 = 0.2-0.8 and c2 = 0.2-0.8, simultaneously decrease - as is typical in the severe non-thyroid illness syndrome. **(E)** The physiologically-based molecular model (32) predicts a similar outcome, and **(F)** the model predictions are clinically observed in a sample of critically ill patients in intensive care (33). For the clinical data in panels **(C, F)**, indicative regression lines with 95% confidence intervals are shown. To permit a comparison across the different models and clinical data, estimates of production rate constant and the levels of FT3 and FT4 are scaled, normalized for each model and normalized mean centered for the data using a euthyroid sample, and expressed as a percentage of the normalized level. Full details are provided in **Supplementary Material**. Color code of hormones: FT4 blue, FT3 green.

Under conditions of decreasing FT4 production (shortage of supply), which describes the onset of slowly progressive autoimmune thyroiditis (Hashimoto’s disease) (31), system (sys. 10) predicts that the level of FT3 and the utilization of FT3 can be maintained in a range close to the normal range despite the decrease in FT4 production rate constant k32 (**Figure 6A**). Suitable parameters were chosen within a certain range (k423 = 0.2 - 0.3, k43 = 0.7 - 0.8, k42 = 0.1 - 0.2) to approximate proportional contributions by the different sources of FT3 according to human physiology (15) and previous modelling (32). We compare the predicted ranges of FT3 and FT4 with those predicted by a physiologically-based molecular model (32) previously validated by clinical data (**Figure 6B**) and the results obtained with a clinical sample of patients with thyroid autoimmune disease (31) (**Figure 6C**). To allow a comparison across different scales and units, outcomes are normalized, mean centered, and expressed as a percentage change. The predictions of the minimal model (**Figure 6A**) are in good agreement with the other model (**Figure 6B**) and the clinical results (**Figure 6C**).

The HPT axis regulation is impaired by severe disease, a condition termed the non-thyroid illness syndrome or thyroid allostasis in critical illness (17). This causes typical changes in multiple system parameters, simultaneously affecting several mechanisms in the HPT axis regulation, particularly decreasing the FT3 production by various organs and increasing the influence of negative feedback by FT4 and FT3 on TSH and TRH (17). This syndrome is clinically characterized by low FT3 levels in the presence of normal FT4 concentrations - although FT4 and TSH may be additionally decreased in more severe cases (17). Our modelling, system (sys. 10) with suitable ranges of some parameters according to pathophysiology (17), namely k423 = 0.2 - 0.3, k43 = 0.1 - 0.4, k42 = 0.1 - 0.2, s1 = 0.2 - 0.8, c2 = 0.2 - 0.8, predicts that the levels of FT3 cannot be maintained within the normal range, and decrease relatively more than FT4 levels (**Figure 5D**). This is in agreement with the predictions of the molecular model (32) (**Figure 6E**) and the results of clinical data from patients with severe non-thyroidal illness in intensive care (33) (**Figure 6F**).

This provides evidence that our minimal model is capable to generate testable predictions about principles and mechanisms of the HPT axis regulation.

## Discussion

Biological systems proceed with great efficiency to achieve their goals, amongst which robustness and maintenances and homeostasis are foremost (54). Using the HPT axis and FT3 homeostasis as an example, we have formulated, as proof-of-concept, a minimal mathematical model, which includes the necessary hierarchical levels to describe the regulatory mechanisms expressed by the HPT axis (**Figure 1**). In a detailed analysis of the system components and mechanisms, we have discovered the system’s essential regulatory principles and properties within this complex network. We have rationalized and integrated the apparently contradictory requirement of robustness to change with the ability of adaptation to new circumstances when appropriate. The ability of the HPT axis to smoothly alter direction from resistance to change to the attainment of a new level of homeostasis demonstrates the considerable flexibility in the system.

Organizational efficiency in achieving homeostasis can emerge from joining elements of top-down and bottom-up regulation (13). Traditionally, many hierarchical models have divorced these two elements of control. Clinical studies have obscured a clear distinction between them by relying on averaged statistical evaluations (18). Consideration of top-down control alone, though most successfully employed in the technical world, tends to be less suitable for the natural world, where the additional important role of bottom-up control has been increasingly recognized (13, 14). In psychology and the neurosciences, a reconsideration has begun with the reformulation of models for predictive coding (13).

Here we examine the integration of top-down and bottom-up regulation in the hierarchy of an endocrine system, namely the HPT axis (15, 17). In particular, our model uncovered critical mechanisms in the HPT axis regulation to support “perfect FT3 homeostasis”. Of note, FT3 homeostasis is preserved by these mechanisms when the thyroidal FT4 production rate varies. Physiologically, preservation of FT3 homeostasis under such conditions is in agreement with clinical data in patients with subclinical hypothyroidism due to autoimmune thyroiditis (**Figure 6**). The prediction was also reproduced with a molecular model (32) using physiological reaction constants as parameters (**Figure 6**).

We further show that, if required under altered conditions, the system has other mechanisms that allow it to adapt to change by shifting the homeostatic level of FT3. The antagonistic properties of the system, namely to preserve FT3 homeostasis or to adapt to a new level, therefore cannot be viewed as unrelated objectives. Rather both contribute to the resilience of the system in a broader sense and provide the system with the flexibility to choose between different mechanisms. Our modeling shows that top-down control is instrumental in delivering optimum homeostasis and robustness in system performance (“perfect homeostasis”). At the same time, bottom-up regulation provides mechanisms of a loss of homeostasis and allostatic adaptation to altered conditions, for instance in severe non-thyroidal illness (low T3 syndrome (17). Such an example has been modelled and compared to a molecular model (32) and clinical data (**Figure 6**).

We propose that the concept of adaptation extends to the diversity among various organs in expressing individual target variables, reflective of their different metabolic needs within the greater totality of the organism. Diversification may be viewed as requiring mechanisms of adaptivity from imposed strict top-level system control (43) and allowing a degree of additional control at the lower level of each organ. This is not to be confused with full independence and autonomy. Generally, response diversity has been reported to dampen the transition between distinct homeostatic levels avoiding abrupt and harmful system-wide fluctuations (55). It may be for those reasons that the mechanisms of decentralized organ control emerged in endocrine regulation during evolution (51). In the human body, many organs are capable to convert T4, which is exclusively produced by the thyroid gland, into its more active sister hormone T3 by means of enzymatic deiodination at the cell membrane or within the cell (56). In the HPT regulatory network, non-thyroidal T3 production is however not a dependent factor. In our model, in the absence of any T3 sources other than the thyroid, HPT axis regulation remains self-sufficient to support FT3 homeostasis. This finding, providing an important rescue mechanism, has been experimentally confirmed in genetically modified animals devoid of T4 to T3 converting deiodinating enzymes (52). Despite this, these animals maintained their homeostatic level in a range appropriate for their developmental and metabolic needs (52).

Because these mechanisms preserve FT3 homeostasis independently of the FT4 production rate constant (k32), this provides the flexibility for the body to store and/or utilize FT4 for its needs without affecting FT3 homeostasis. The findings provide a plausible mechanism for the anticipatory actions of the HPT axis regulation as found in animal studies (17). This is consistent with the system’s defensive response in anticipation of and preparation for a perceived threat without a prior need to experience the harmful impact on the body.

Importantly, the network of the HPT axis regulation has the flexibility to discriminate between unwanted homeostatic perturbations and a directed response to a stress challenge, expressing joint properties of preservation of homeostasis and adaptation. Unlike in engineered systems (57), complexity avoidance is not a necessary attribute for adaptability in natural systems. These, such as the HPT axis, are able to maintain or precisely adjust a homeostatic level of a target physiological variable in the face of naturally occurring variations in other reactions of the system components. This includes genetic and environmental variations in the expression of the setpoint (FT4 and TSH equilibrium point) (58). To this end, a host of neuronal and humoral signals impacts the TRH neuron, either directly at the paraventricular nucleus or through close interaction with neighboring tancytes (49, 50). A change of TRH, in turn, shifts the homeostatic level of FT3, which must be increased or decreased in a precise and accurate manner to adjust (fine-tune) the metabolic rate. This applies to different environmental conditions, such as seasonal change, cold exposure, fasting, weight gain, infectious diseases, and psychological stress (17). Our model shows that the increased complexity at the upper level does not necessarily result in a loss of stability and precision at the lower level, with the system continuously integrating and translating upper-level influences into a sensitively and precisely adjusted homeostatic outcome.

The model uncovers the properties of system resilience to cope with various forms of stress. This can be achieved through a flexible combination of mechanisms critical for homeostasis and adaptation of, in our case, FT3 in the HPT axis regulation. In cell circuits, components of antagonistic regulation, exerting simultaneous opposing effects on the same target, have been identified to support robustness (34). Such components allow for a system response that resists fluctuations in a precursor or initial amount (34). This is a physiologically desirable mechanism shared by our minimal HPT axis model (eq. (1.4)). Coupled positive-negative feedback loops have been generally suggested to support switching between multiple homeostatic states (11). System resilience has received increasing consideration in the emerging field of metabolic network designs (59). While there is no universal definition of resilience, patterns of resilience have been discussed for different systems (60). While the thyroid stress response is less dynamic, recent insights into the stress response of the hypothalamic-pituitary-adrenal (HPA) axis have linked chronic disruption of circadian rhythms to the accumulation of allostatic load (61, 62). Adaptation of the HPA axis has been modeled as a trade-off between its stress-responsiveness and its time-keeping ability, and individualized allostatic adaptation strategies have emerged (63).

The minimal model of HPT regulation provides fundamental insights into the principles of effective system control, revealing several favorable design properties. These comprise 1) FT3 homeostasis, 2) extended independence of FT3 from its precursor FT4, 3) protection of T3 utilization against a shortage in T4 supply, 4) protection against a potentially harmful over-shooting FT3 response, 5) anticipatory corrective action (resetting) by the central level before experiencing perturbations at the corporeal sites. Together, these properties allow the system to sensitively and pro-actively react to challenges and smoothly adapt to varying circumstances while retaining at the same time the necessary robustness to avoid major perturbations of the homeostatic variable.

FT3 homeostasis is closely related to the energy homeostasis of the human body (12), particularly basal metabolic rate and resting energy expenditure (64, 65). FT3 homeostasis and adaptation is a major goal of the HPT regulation in our model, subject to attainment of the joint implementation of the described mechanisms of top-down and bottom-up regulation for its attainment. This perspective contrasts with models such as the classical model which confines itself to the top-down control of FT4 production by TSH, as currently used in diagnostic guidelines (66). Here, TSH is used as a universal marker of thyroid function to biochemically define the thyroid diseases of hypo- and hyperthyroidism including subclinical hypothyroidism/hyperthyroidism without a clinical correlate (66). However, the traditional understanding remains incomplete and unsatisfactory for the following reasons: 1) not considering the essential role of FT3 homeostasis in the production and utilization of thyroid hormones, 2) not integrating supply (FT4 production) and demand (FT3 requirements), 3) not considering FT3-feedback onto TSH and TSH-feedforward onto FT3, divorcing top-down and bottom-up regulation in the HPT axis regulation, and 4) not considering joint mechanisms for FT3 homeostasis of the hypothalamus, pituitary, and peripheral organs.

At the onset of a hypothyroid or hyperthyroid state, our model does not assign TSH a role as a disease marker, which passively reflects the thyroid functional state. Rather it gives it a corrective role as a pro-active regulator of thyroid function, which aims to preserve FT3 homeostasis despite a decline in its precursor FT4. This has important implications for the diagnosis and treatment of such patients, especially those who cannot adequately process T4-T3 conversion in the absence of a viable thyroid (45). In this respect, our model challenges the traditional view (66), as do recent clinical studies (67).

In our proof-of-concept mathematical model, as a limitation, physiologically realistic time-dependencies and the influence of numerous potentially interfering variabilities and modifying real-world influences have not been detailed. The latter is beyond the aim and scope of this article and capability of the minimal model. We refer the reader to some physiologically realistic models constructed phenomenologically by our group and others (32, 37–39, 68). Notwithstanding these practical limitations, we describe a reconciliation of two antagonistic mechanisms in a multivariable regulatory network of the HPT axis. This supports a current homeostatic state and producing adaptation (allostasis) by shifting the current homeostatic state to a new one under varying circumstances. A combination of these two mechanisms provides considerable regulatory flexibility to choose between them as appropriate in different physiological conditions. The new theoretical insights into the mechanisms of effective system control of the HPT axis detail principles of endocrine regulation. We propose that this can be applied to both hypothesis-testing in the HPT regulation and, likely, other endocrine systems of similar structure.

## Data Availability Statement

The original contributions presented in the study are included in the article/**Supplementary Material**. Further inquiries can be directed to the corresponding author.

## Author Contributions

Conceptualization: RH, MP, JM, RL, JD. Methodology: RH, MP, JD. Investigation: MP, RH. Visualization: RH. Writing - original draft: RH, MP. Writing - review& editing: RH, MP, JM, RL, JD. All authors contributed to the article and approved the submitted version.

## Conflict of Interest

JD is co-owner of the intellectual property rights for the patent “System and Method for Deriving Parameters for Homeostatic Feedback Control of an Individual” (Singapore Institute for Clinical Sciences, Biomedical Sciences Institutes, Application Number 201208940-5, WIPO number WO/2014/088516).

All remaining authors declare that the research was conducted in the absence of any commercial or financial relationships that could be construed as a potential conflict of interest.

## Publisher’s Note

All claims expressed in this article are solely those of the authors and do not necessarily represent those of their affiliated organizations, or those of the publisher, the editors and the reviewers. Any product that may be evaluated in this article, or claim that may be made by its manufacturer, is not guaranteed or endorsed by the publisher.
