# Supplementary material for: Principles of Endocrine Regulation: Reconciling Tensions Between Robustness in Performance and Adaptation to Change
Source: Front Endocrinol (Lausanne). 2022 Jun 9;13:825107. doi: 10.3389/fendo.2022.825107 (PMC9219553; doi:10.3389/fendo.2022.825107)
Supplement: Supplementary file 1 [file DataSheet_1.pdf]

Supplementary Material

Principles of Endocrine Regulation: Reconciling Tensions between Robustness in Performance and Adaptation to Change

Rudolf Hoermann*, Mark J. Pekker (formerly Mark Friedman), John E. M. Midgley, Rolf Larisch and Johannes W. Dietrich

*Correspondence: rudolf.hoermann@gmail.com

Supplementary Code and Data

Minimal model of the hypothalamic-pituitary-thyroid (HPT) axis regulation

Mechanisms of FT3 homeostasis

Version 1.0 by RH

Authors: R. Hoermann, M.J. Pekker, J.E.M. Midgley, R. Larisch, J.W. Dietrich

Minimal model

ODEs of system (1)

$$\frac{d[TRH]}{dt} = \frac{s_1}{k_{134} \cdot [FT_4] \cdot [FT_3]} - a_1 \cdot [TRH] \quad \text{(eq. 1.1)}$$

$$\frac{d[TSH]}{dt} = k_{21} \cdot [TRH] + \frac{c_2}{k_{234} \cdot [FT_4] \cdot [FT_3])} - a_2 \cdot [TSH] \quad \text{(eq. 1.2)}$$

$$\frac{d[FT_4]}{dt} = k_{32} \cdot [TSH] - a_3 \cdot [FT_4] \quad \text{(eq. 1.3)}$$

$$\frac{d[FT_3]}{dt} = k_{423} \cdot [TSH] \cdot [FT_4] - a_4 \cdot [FT_3] \quad \text{(eq. 1.4)}$$

# analytical equilibrium solutions of system (1)

$$[TRH] = a_2 \cdot \sqrt{a}_3 \cdot \sqrt{a}_4 \cdot s_1 \cdot k_{234} \cdot \sqrt{\frac{\sqrt{k}_{423} \cdot \sqrt{a_1 \cdot c_2 \cdot k_{134} + s1 \cdot k_{21} \cdot k_{234}}}{\sqrt{a}_1 \cdot \sqrt{a}_2 \cdot \sqrt{a}_4 \cdot \sqrt{k}_{134} \cdot \sqrt{k}_{234}}} \cdot \frac{1}{\sqrt{k}_{32} \cdot \sqrt{k}_{423} \cdot (a_1 \cdot c_2 \cdot k_{134} + s_1 \cdot k_{21} \cdot k_{234})}$$

$$[TSH] = \sqrt{\sqrt{a}_3 \cdot \sqrt{a}_4 \cdot \frac{\sqrt{k}_{423} \cdot \sqrt{(a_1 \cdot c_2 \cdot k_{134} + s_1 \cdot k_{21} \cdot k_{234})}}{\sqrt{a}_1 \cdot \sqrt{a}_2 \cdot \sqrt{a}_4 \cdot \sqrt{k}_{134} \cdot \sqrt{k}_{234}} \cdot \frac{1}{\sqrt{k}_{32} \cdot \sqrt{k}_{423}}}$$

$$[FT4] = \sqrt{\sqrt{a}_4 \cdot \sqrt{k}_{32} \cdot \frac{\sqrt{k}_{423} \cdot \sqrt{(a_1 \cdot c_2 \cdot k_{134} + s_1 \cdot k_{21} \cdot k_{234})}}{\sqrt{a}_1 \cdot \sqrt{a}_2 \cdot \sqrt{a}_4 \cdot \sqrt{k}_{134} \cdot \sqrt{k}_{234}} \cdot \frac{1}{\sqrt{a}_3 \cdot \sqrt{k}_{423}}}$$

$$[FT_3] = \sqrt{\frac{k_{423} \cdot (a_1 \cdot c_2 \cdot k_{134} + s1 \cdot k_{21} \cdot k_{234})}{a_1 \cdot a_2 \cdot a_4 \cdot k_{134} \cdot k_{234}}}$$

R scripts to solve the ODE systems of the model and generate the figures

Below scripts are provided - in addition to the more accurate analytical solutions reported in the paper - to enable interested researchers to reproduce the mathematical model in the free R environment (1), which can quickly solve the ODE system and conveniently display the solutions in a versatile way. We used R 4.1.2 for Mac (1), which is freely available from the R Core Team 2021 at https://www.R-project.org/.

We expect potential users to be generally familiar with the R programming language, understand the coding sequence and require them to have the following specific packages and their dependencies installed, FME (2), deSolve (3), and rootSolve (3). For arranging the panels, we used the packages ggplot2 (4), ggplotify (5) and patchworks (6).

References

1. R Core Team. R: A language and environment for statistical computing. R Foundation for Statistical Computing. 2020;ISBN 3-900051-07-0. Available from https://www.R-project.org/.

2. Soetaert K, Petzoldt T. Inverse modelling, sensitivity and Monte Carlo analysis in R using package FME. J Stat Softw. 2010;33. doi:10.18637/jss.v033.i03

3. Soetaert K, Herman PMJ. A practical guide to ecological modelling. 2009:1-372. doi:10.1007/978-1-4020-8624-3

4. H. Wickham. ggplot2: Elegant Graphics for Data Analysis. Springer-Verlag New York, 2016. Available from https://ggplot2.tidyverse.org.

5. Yu G. ggplotify: Convert plot to 'grob' or 'ggplot' object. R package version 0.1.0. 2021. Available from https://CRAN.R-project.org/package=ggplotify.

6. Pedersen TL. patchwork: The composer of plots. R package version 1.1.1. 2020. Available from https://CRAN.R-project.org/package=patchwork.

Scripts to generate the graphical solutions and figures

```
# system (1)
fn1=function(t,y,k){
with (as.list(c(y,k)), {
dTRH= s1/(k134*FT4*FT3) - a1*TRH
dTSH= k21*TRH+c2/(k234*FT4*FT3) - a2*TSH
dFT4=  k32*TSH - a3*FT4
dFT3=   k423*TSH*FT4 - a4*FT3
return(list(c(dTRH,dTSH,dFT4,dFT3)))
})
}
# system parameters
k=c(s1=1,k134=1,k21=1,c2=1,k234=1,k32=1,k423=1,a1=1,a2=1,a3=1,a4=1)
# initial values
y0=c(TRH=1,TSH=1,FT4=1,FT3=1)
# time-dependent solution
times=seq(0,20, by=1)
out1=ode(y=y0,func=fn1,times=seq(0,20,by=1),parms=k)
plot(out1)
# function to find the equilibrium solution and vary selected parameters
fs2= function(p) {
s1 <- steady(y=y0,func=fn1,parms=p,method="runsteady")
return(data.frame(TRH=s1$y[1],TSH=s1$y[2],FT4=s1$y[3],FT3=s1$y[4]))
}
```

```
# plot
plot(out1)
library(ggplotify)
p2a=as.ggplot(~plot(out1))
p2a
# vary k32 within a range
parRanges <- data.frame(min = c(0.3), max = c(3));
rownames(parRanges) <- c("k32");
# find equilibrium solutions
r1 <- sensRange(func=fs2,parms = k,parRange=parRanges,
sensvar=c("FT4","FT3","TSH","TRH"),num=1000);
# plot
p2b=ggplot() +
geom_point(aes(x = k32,y = FT3),data=r1,colour = '#33ff00',size = 0.5) +
geom_point(aes(x = k32,y = FT4),data=r1,colour = '#0066ff',size = 0.5) +
geom_point(aes(x = k32,y = TSH),data=r1,size = 0.5) +
geom_point(aes(x = k32,y = TRH),data=r1,colour = '#ff9900',size = 0.5) +
theme_classic() +
ylab(label = 'TRH TSH FT4 FT3')+
ylim(0,3);
p2b
# system (1 mod)
# replace (eq. 1.2) with (eq. 1.2 mod)
dTSH= k21*TRH*c2/(k234*FT4*FT3) - a2*TSH     (eq. 1.2 mod)
y0=c(TRH=0.9,TSH=1,FT4=1,FT3=1)
# specify k21 and the range of k32
parRanges <- data.frame(min = c(2,0.3), max = c(2,3));
rownames(parRanges) <- c("k21","k32");
# find equilibrium solution
r1 <- sensRange(func=fs2,parms = k,parRange=parRanges,
sensvar=c("FT4","FT3","TSH","TRH"),num=1000);
```

```
# plot
p2c=ggplot() +
geom_point(aes(x = k32,y = FT3),data=r1,colour = '#33ff00',size = 0.5) +
geom_point(aes(x = k32,y = FT4),data=r1,colour = '#0066ff',size = 0.5) +
geom_point(aes(x = k32,y = TSH),data=r1,size = 0.5) +
geom_point(aes(x = k32,y = TRH),data=r1,colour = '#ff9900',size = 0.5) +
theme_classic() +
ylab(label = 'TRH TSH FT4 FT3')+
ylim(0,3);
p2c
# Fig. 2, arrange the panels
library(patchwork)
p2=p2a + p2b + p2c + plot_annotation(tag_levels = 'A') + plot_layout(nrow= 2, byrow=TRUE)
p2


# Multiple systems (for derivation and details see paper)
# system (1)
fn1=function(t,y,k){
with (as.list(c(y,k)), {
dTRH= s1/(k134*FT4*FT3) - a1*TRH
dTSH= k21*TRH+c2/(k234*FT4*FT3) - a2*TSH
dFT4=  k32*TSH - a3*FT4
dFT3=   k423*TSH*FT4 - a4*FT3
return(list(c(dTRH,dTSH,dFT4,dFT3)))
})
}
k=c(s1=1,k134=1,k21=1,c2=1,k234=1,k32=1,k423=1,a1=1,a2=1,a3=1,a4=1)
y0=c(TRH=1,TSH=1,FT4=1,FT3=1)
parRanges <- data.frame(min = c(0.3), max = c(3));
rownames(parRanges) <- c("k32");
```

```
r1 <- sensRange(func=fs2,parms = k,parRange=parRanges,
sensvar=c("FT4","FT3","TSH","TRH"),num=1000);
# system (2)
fn1=function(t,y,k){
with (as.list(c(y,k)), {
dTRH= s1/(k134*1*1) - a1*TRH
dTSH= k21*TRH+c2/(k234*FT4*FT3) - a2*TSH
dFT4=  k32*TSH - a3*FT4
dFT3=   k423*TSH*FT4 - a4*FT3
return(list(c(dTRH,dTSH,dFT4,dFT3)))
})
}
k=c(s1=1,k134=1,k21=1,c2=1,k234=1,k32=1,k423=1,a1=1,a2=1,a3=1,a4=1)
parRanges <- data.frame(min = c(0.3), max = c(3));
rownames(parRanges) <- c("k32");
r2 <- sensRange(func=fs2,parms = k,parRange=parRanges,
sensvar=c("FT4","FT3","TSH","TRH"),num=1000);
# system (3)
fn1=function(t,y,k){
with (as.list(c(y,k)), {
dTRH= s1/(k134*FT4*FT3) - a1*TRH
dTSH= k21*TRH+c2/(k234*1*1) - a2*TSH
dFT4=  k32*TSH - a3*FT4
dFT3=   k423*TSH*FT4 - a4*FT3
return(list(c(dTRH,dTSH,dFT4,dFT3)))
})
}
k=c(s1=1,k134=1,k21=1,c2=1,k234=1,k32=1,k423=1,a1=1,a2=1,a3=1,a4=1)
parRanges <- data.frame(min = c(0.3), max = c(3));
rownames(parRanges) <- c("k32");
```

```
r3 <- sensRange(func=fs2,parms = k,parRange=parRanges,
sensvar=c("FT4","FT3","TSH","TRH"),num=1000);
# system (8)
fn1=function(t,y,k){
with (as.list(c(y,k)), {
dTRH= s1/(k134*FT4*FT3) - a1*TRH
dTSH= k21*TRH+c2/(k234*FT4*FT3) - a2*TSH
dFT4= k32*TSH - a3*FT4
dFT3=  k423*1*FT4 - a4*FT3
return(list(c(dTRH,dTSH,dFT4,dFT3)))
})
}
k=c(s1=1,k134=1,k21=1,c2=1,k234=1,k32=1,k423=1,a1=1,a2=1,a3=1,a4=1)
parRanges <- data.frame(min = c(0.3), max = c(3));
rownames(parRanges) <- c("k32");
r4 <- sensRange(func=fs2,parms = k,parRange=parRanges,
sensvar=c("FT4","FT3","TSH","TRH"),num=1000);
# plot
p3a=ggplot() +
geom_point(aes(x = k32,y = FT3),data=r1,colour = '#33ff00',size = 0.5) +
geom_point(aes(x = k32,y = FT3),data=r2,colour = 'darkgreen',size = 0.5) +
geom_point(aes(x = k32,y = FT3),data=r3,colour = 'darkgreen',size = 0.5) +
geom_point(aes(x = k32,y = FT3),data=r4,colour = '#CAFF70',size = 0.5) +
theme_classic() +
ylab(label='FT3')+
ylim(0,4)
p3a
# Multiple systems (for derivation and details see paper)
# system (1)
rm(out1,s1)
fn1=function(t,y,k){
```

```
with (as.list(c(y,k)), {
dTRH= s1/(k134*FT4*FT3) - a1*TRH
dTSH= k21*TRH+c2/(k234*FT4*FT3) - a2*TSH
dFT4=  k32*TSH - a3*FT4
dFT3=   k423*TSH*FT4 - a4*FT3
return(list(c(dTRH,dTSH,dFT4,dFT3)))
})
}
k=c(s1=1,k134=1,k21=1,c2=1,k234=1,k32=1,k423=1,a1=1,a2=1,a3=1,a4=1)
parRanges <- data.frame(min = c(0.3), max = c(3));
rownames(parRanges) <- c("k32");
r1 <- sensRange(func=fs2,parms = k,parRange=parRanges,
sensvar=c("FT4","FT3","TSH","TRH"),num=1000);
# system (4)
fn1=function(t,y,k){
with (as.list(c(y,k)), {
dTRH= s1/(k134*FT4*1) - a1*TRH
dTSH= k21*TRH+c2/(k234*FT4*FT3) - a2*TSH
dFT4=  k32*TSH - a3*FT4
dFT3=   k423*TSH*FT4 - a4*FT3
return(list(c(dTRH,dTSH,dFT4,dFT3)))
})
}
k=c(s1=1,k134=1,k21=1,c2=1,k234=1,k32=1,k423=1,a1=1,a2=1,a3=1,a4=1)
parRanges <- data.frame(min = c(0.3), max = c(3));
rownames(parRanges) <- c("k32");
r4 <- sensRange(func=fs2,parms = k,parRange=parRanges,
sensvar=c("FT4","FT3","TSH","TRH"),num=1000);
# system (5)
fn1=function(t,y,k){
with (as.list(c(y,k)), {
```

```
dTRH= s1/(k134*1*FT3) - a1*TRH

dTSH= k21*TRH+c2/(k234*FT4*FT3) - a2*TSH

dFT4=  k32*TSH - a3*FT4

dFT3=   k423*TSH*FT4 - a4*FT3

return(list(c(dTRH,dTSH,dFT4,dFT3)))

})

}

k=c(s1=1,k134=1,k21=1,c2=1,k234=1,k32=1,k423=1,a1=1,a2=1,a3=1,a4=1)

# vary parameters

parRanges <- data.frame(min = c(0.3), max = c(3));

rownames(parRanges) <- c("k32");

r5 <- sensRange(func=fs2,parms = k,parRange=parRanges,

sensvar=c("FT4","FT3","TSH","TRH"),num=1000);

# system (6)

fn1=function(t,y,k){

with (as.list(c(y,k)), {

dTRH= s1/(k134*FT4*FT3) - a1*TRH

dTSH= k21*TRH+c2/(k234*FT4*1) - a2*TSH

dFT4=  k32*TSH - a3*FT4

dFT3=   k423*TSH*FT4 - a4*FT3

return(list(c(dTRH,dTSH,dFT4,dFT3)))

})

}

k=c(s1=1,k134=1,k21=1,c2=1,k234=1,k32=1,k423=1,a1=1,a2=1,a3=1,a4=1)

parRanges <- data.frame(min = c(0.3), max = c(3));

rownames(parRanges) <- c("k32");

r6 <- sensRange(func=fs2,parms = k,parRange=parRanges,

sensvar=c("FT4","FT3","TSH","TRH"),num=1000);

# system (7)

fn1=function(t,y,k){
```

```
with (as.list(c(y,k)), {
dTRH= s1/(k134*FT4*FT3) - a1*TRH
dTSH= k21*TRH+c2/(k234*1*FT3) - a2*TSH
dFT4=  k32*TSH - a3*FT4
dFT3=   k423*TSH*FT4 - a4*FT3
return(list(c(dTRH,dTSH,dFT4,dFT3)))
})
}
k=c(s1=1,k134=1,k21=1,c2=1,k234=1,k32=1,k423=1,a1=1,a2=1,a3=1,a4=1)
parRanges <- data.frame(min = c(0.3), max = c(3));
rownames(parRanges) <- c("k32");
r7 <- sensRange(func=fs2,parms = k,parRange=parRanges,
sensvar=c("FT4","FT3","TSH","TRH"),num=1000);
# plot
p3b=ggplot() +
geom_point(aes(x = k32,y = FT3),data=r1,colour = '#33ff00',size = 0.5) +
geom_point(aes(x = k32,y = FT3),data=r4,colour = 'darkgreen',size = 0.5) +
geom_point(aes(x = k32,y = FT3),data=r5,colour = '#CAFF70',size = 0.5) +
geom_point(aes(x = k32,y = FT3),data=r6,colour = 'darkgreen',size = 0.5) +
geom_point(aes(x = k32,y = FT3),data=r7,colour = '#CAFF70',size = 0.5) +
theme_classic() +
ylab(label='FT3')+
ylim(0,3)
p3b
# Draw Fig. 3, arrange the panels
p3=p3a + p3b + plot_annotation(tag_levels = 'A') + plot_layout(nrow= 1, byrow=TRUE)
p3


# Multiple systems (for derivation and details see paper)
fn1=function(t,y,k){
with (as.list(c(y,k)), {
```

```
dTRH= s1/(k134*FT4*FT3) - a1*TRH
dTSH= k21*TRH+c2/(k234*FT4*FT3) - a2*TSH
dFT4=  k32*TSH + drug4 - a3*FT4
dFT3= k423*TSH*FT4 + k43*FT4 + k42*TSH + drug3 - a4*FT3
return(list(c(dTRH,dTSH,dFT4,dFT3)))
})
}
k=c(s1=1,k134=1,k21=1,c2=1,k234=1,k32=1,drug4=0,k423=1,k43=1,k42=0,drug3=0,a1=1,a2=1,a3=1,a
4=1)
parRanges <- data.frame(min = c(0.3,1,0,0,0,0), max = c(3,1,0,0,0,0));
rownames(parRanges) <- c("k32","k423","k43","k42","drug4","drug3");
parRanges
rm(r1)
r1 <- sensRange(func=fs2,parms = k,parRange=parRanges,
sensvar=c("FT4","FT3","TSH","TRH"),num=1000);
p4a=ggplot() +
geom_point(aes(x = k32,y = FT3),data=r1,colour = '#33ff00',size = 0.5) +
geom_point(aes(x = k32,y = FT4),data=r1,colour = '#0066ff',size = 0.5) +
geom_point(aes(x = k32,y = TSH),data=r1,size = 0.5) +
theme_classic() +
geom_point(aes(x = k32,y = TRH),data=r1,colour = '#ff9900',size = 0.5) +
ylab(label = 'TRH TSH FT4 FT3')+
ylim(0,3)
p4a
parRanges <- data.frame(min = c(0.3,1,1,0,0,0), max = c(3,1,1,0,0,0));
rownames(parRanges) <- c("k32","k423","k43","k42","drug4","drug3");
parRanges
r1 <- sensRange(func=fs2,parms = k,parRange=parRanges,
sensvar=c("FT4","FT3","TSH","TRH"),num=1000);
p4b=ggplot() +
geom_point(aes(x = k32,y = FT3),data=r1,colour = '#33ff00',size = 0.5) +
```

```
geom_point(aes(x = k32,y = FT4),data=r1,colour = '#0066ff',size = 0.5) +
geom_point(aes(x = k32,y = TSH),data=r1,size = 0.5) +
theme_classic() +
geom_point(aes(x = k32,y = TRH),data=r1,colour = '#ff9900',size = 0.5) +
ylab(label = 'TRH TSH FT4 FT3')+
ylim(0,3)
p4b
parRanges <- data.frame(min = c(0.3,1,1,1,0,0), max = c(3,1,1,1,0,0));
rownames(parRanges) <- c("k32","k423","k43","k42","drug4","drug3");
parRanges
r1 <- sensRange(func=fs2,parms = k,parRange=parRanges,
sensvar=c("FT4","FT3","TSH","TRH"),num=1000);
p4c=ggplot() +
geom_point(aes(x = k32,y = FT3),data=r1,colour = '#33ff00',size = 0.5) +
geom_point(aes(x = k32,y = FT4),data=r1,colour = '#0066ff',size = 0.5) +
geom_point(aes(x = k32,y = TSH),data=r1,size = 0.5) +
geom_point(aes(x = k32,y = TRH),data=r1,colour = '#ff9900',size = 0.5) +
theme_classic() +
ylab(label = 'TRH TSH FT4 FT3')+
ylim(0,3)
p4c
# T3 drug
parRanges <- data.frame(min = c(0.3,1,1,1,0.5), max = c(3,1,1,1,0.5));
rownames(parRanges) <- c("k32","k423","k43","k42","drug3");
r1 <- sensRange(func=fs2,parms = k,parRange=parRanges,
sensvar=c("FT4","FT3","TSH","TRH"),num=1000);
p4d=ggplot() +
geom_point(aes(x = k32,y = FT3),data=r1,colour = '#33ff00',size = 0.5) +
geom_point(aes(x = k32,y = FT4),data=r1,colour = '#0066ff',size = 0.5) +
geom_point(aes(x = k32,y = TSH),data=r1,size = 0.5) +
geom_point(aes(x = k32,y = TRH),data=r1,colour = '#ff9900',size = 0.5) +
```

```
theme_classic() +
ylab(label = 'TRH TSH FT4 FT3')+
ylim(0,4)
p4d
# T4 drug
parRanges <- data.frame(min = c(0.3,1,1,1,0.8), max = c(3,1,1,1,0.8));
rownames(parRanges) <- c("k32","k423","k43","k42","drug4");
r1 <- sensRange(func=fs2,parms = k,parRange=parRanges,
sensvar=c("FT4","FT3","TSH","TRH"),num=1000);
p4e=ggplot() +
geom_point(aes(x = k32,y = FT3),data=r1,colour = '#33ff00',size = 0.5) +
geom_point(aes(x = k32,y = FT4),data=r1,colour = '#0066ff',size = 0.5) +
geom_point(aes(x = k32,y = TSH),data=r1,size = 0.5) +
geom_point(aes(x = k32,y = TRH),data=r1,colour = '#ff9900',size = 0.5) +
theme_classic() +
ylab(label = 'TRH TSH FT4 FT3')+
ylim(0,4)
p4e
# T3 T4 drug
parRanges <- data.frame(min = c(0.3,1,1,1,0.8,0.5), max = c(3,1,1,1,0.8,0.5));
rownames(parRanges) <- c("k32","k423","k43","k42","drug4","drug3");
r1 <- sensRange(func=fs2,parms = k,parRange=parRanges,
sensvar=c("FT4","FT3","TSH","TRH"),num=1000);
p4f=ggplot() +
geom_point(aes(x = k32,y = FT3),data=r1,colour = '#33ff00',size = 0.5) +
geom_point(aes(x = k32,y = FT4),data=r1,colour = '#0066ff',size = 0.5) +
geom_point(aes(x = k32,y = TSH),data=r1,size = 0.5) +
geom_point(aes(x = k32,y = TRH),data=r1,colour = '#ff9900',size = 0.5) +
theme_classic() +
ylab(label = 'TRH TSH FT4 FT3')+
ylim(0,5)
```

```
p4f
# modify s1
parRanges <- data.frame(min = c(0.3,1,0,0,1.5), max = c(3,1,0,0,1.5));
rownames(parRanges) <- c("k32","k423","k43","k42","s1");
parRanges
r1 <- sensRange(func=fs2,parms = k,parRange=parRanges,
sensvar=c("FT4","FT3","TSH","TRH"),num=1000);
p4g=ggplot() +
geom_point(aes(x = k32,y = FT3),data=r1,colour = '#33ff00',size = 0.5) +
geom_point(aes(x = k32,y = FT4),data=r1,colour = '#0066ff',size = 0.5) +
geom_point(aes(x = k32,y = TSH),data=r1,size = 0.5) +
theme_classic() +
geom_point(aes(x = k32,y = TRH),data=r1,colour = '#ff9900',size = 0.5) +
ylab(label = 'TRH TSH FT4 FT3')+
ylim(0,3)
p4g
# Fig. 4, arrange the panels
p4=p4a + p4b + p4c + p4d + p4e + p4f + p4g + plot_annotation(tag_levels = 'A') + plot_layout(nrow= 3,
byrow=TRUE)
p4


# Multiple systems (for derivation and details see paper)
# system (9)	equals system (10) with k42=0
fn1=function(t,y,k){
with (as.list(c(y,k)), {
dTRH= s1/(k134*FT4*FT3) - a1*TRH
dTSH= k21*TRH+c2/(k234*FT4*FT3) - a2*TSH
dFT4=  k32*TSH - a3*FT4
dFT3= k423*TSH*FT4 + k43*FT4 + k42*TSH - a4*FT3
return(list(c(dTRH,dTSH,dFT4,dFT3)))
})
```

```
}
k=c(s1=1,k134=1,k21=1,c2=1,k234=1,k32=1,k423=1,k43=1,k42=1,a1=1,a2=1,a3=1,a4=1)
y0=c(TRH=1,TSH=1,FT4=1,FT3=1)
parRanges <- data.frame(min = c(1,0.5,0,0), max = c(1,0.5,1,0));
rownames(parRanges) <- c("k32","k423","k43","k42");
r1 <- sensRange(func=fs2,parms = k,parRange=parRanges,
sensvar=c("FT4","FT3","TSH","TRH"),num=1000);
# plot
p5a=ggplot() +
geom_point(aes(x = k43,y = FT3),data=r1,colour = '#33ff00',size = 0.5) +
geom_point(aes(x = k43,y = FT4),data=r1,colour = '#0066ff',size = 0.5) +
# geom_point(aes(x = k43,y = TSH),data=r1,size = 0.5) +
# geom_point(aes(x = k43,y = TRH),data=r1,colour = '#ff9900',size = 0.5) +
theme_classic() +
ylab(label = 'FT4 FT3')+
ylim(0,2)
p5a
# system (9)      equals system (10) with k42=0
parRanges <- data.frame(min = c(0.5,0.5,0,0), max = c(1.5,0.5,1,0));
rownames(parRanges) <- c("k32","k423","k43","k42");
r1 <- sensRange(func=fs2,parms = k,parRange=parRanges,
sensvar=c("FT4","FT3","TSH","TRH"),num=1000);
p5b=ggplot() +
geom_point(aes(x = k32,y = FT3),data=r1,colour = '#33ff00',size = 0.5) +
geom_point(aes(x = k32,y = FT4),data=r1,colour = '#0066ff',size = 0.5) +
geom_point(aes(x = k32,y = TSH),data=r1,size = 0.5) +
# geom_point(aes(x = k32,y = TRH),data=r1,colour = '#ff9900',size = 0.5) +
theme_classic() +
ylab(label = 'TSH FT4 FT3')+
ylim(0,3)
p5b
```

```
# system (10)    with k42=1
parRanges <- data.frame(min = c(0.5,0.5,0,1), max = c(1.5,0.5,1,1));
rownames(parRanges) <- c("k32","k423","k43","k42");
r1 <- sensRange(func=fs2,parms = k,parRange=parRanges,
sensvar=c("FT4","FT3","TSH","TRH"),num=1000);
p5c=ggplot() +
geom_point(aes(x = k32,y = FT3),data=r1,colour = '#33ff00',size = 0.5) +
geom_point(aes(x = k32,y = FT4),data=r1,colour = '#0066ff',size = 0.5) +
geom_point(aes(x = k32,y = TSH),data=r1,size = 0.5) +
theme_classic() +
ylab(label = 'TSH FT4 FT3')+
ylim(0,3)
p5c
# system(10)     with k42=1
parRanges <- data.frame(min = c(0.5,0.5,0,1,0.1), max = c(1.5,0.5,1,1,0.5));
rownames(parRanges) <- c("k32","k423","k43","k42","s1");
r1 <- sensRange(func=fs2,parms = k,parRange=parRanges,
sensvar=c("FT4","FT3","TSH","TRH"),num=1000);
p5d=ggplot() +
geom_point(aes(x = k32,y = FT3),data=r1,colour = '#33ff00',size = 0.5) +
geom_point(aes(x = k32,y = FT4),data=r1,colour = '#0066ff',size = 0.5) +
geom_point(aes(x = k32,y = TSH),data=r1,size = 0.5) +
# geom_point(aes(x = k32,y = TRH),data=r1,colour = '#ff9900',size = 0.5) +
theme_classic() +
ylab(label = 'TSH FT4 FT3')+
ylim(0,3)
p5d
# Fig. 5, arrange panels
p5=p5a + p5b + p5c + p5d + plot_annotation(tag_levels = 'A') + plot_layout(nrow= 2, byrow=TRUE)
p5
```

```
# Multiple systems (for derivation and details see paper)
# system (10)     vary several parameters simultaneously
fn1=function(t,y,k){
with (as.list(c(y,k)), {
dTRH= s1/(k134*FT4*FT3) - a1*TRH
dTSH= k21*TRH+c2/(k234*FT4*FT3) - a2*TSH
dFT4=  k32*TSH - a3*FT4
dFT3= k423*TSH*FT4 + k43*FT4 + k42*TSH - a4*FT3
return(list(c(dTRH,dTSH,dFT4,dFT3)))
})
}
k=c(s1=1,k134=1,k21=1,c2=1,k234=1,k32=1,k423=0.7,k43=0.2,k42=0.1,a1=1,a2=1,a3=1,a4=1)
parRanges <- data.frame(min = c(0.4,0.2,0.7,0.1), max = c(1.1,0.3,0.8,0.2));
rownames(parRanges) <- c("k32","k423","k43","k42");
r1 <- sensRange(func=fs2,parms = k,parRange=parRanges,
sensvar=c("FT4","FT3","TSH","TRH"),num=1000);
# plot
p6a=ggplot() +
geom_point(aes(x = k32*100,y = FT3/1.41*100),data=r1,colour = '#33ff00',size = 0.5) +
geom_point(aes(x = k32*100,y = FT4/1.19*100),data=r1,colour = '#0066ff',size = 0.5) +
#geom_point(aes(x = k32*100,y = TSH/1.19*100),data=r1,size = 0.5) +
theme_classic() +
xlab(label='Estimate of FT4 Production Rate Constant (%)') +
ylab(label='FT4 FT3  (%)') +
ylim(50,150)
p6a
# Draw Fig. 6b, using the model from Hoermann et al. 2020 plus code in Supplementary Material,
Hoermann R el al. Triiodothyronine secretion in early thyroid failure: The adaptive response of central
feedforward control. Eur J Clin Invest. 2020;50:e13192. doi:10.1111/eci.13192

# Draw Fig. 6c, deidentified data are available from Hoermann et al. 2016
```

Hoermann R, Midgley JEM, Larisch R, Dietrich JW. Relational stability of thyroid hormones in euthyroid subjects and patients with autoimmune thyroid disease. Eur Thyroid J. 2016;5:171-179. doi:10.1159/000447967

GT # estimated maximum secretory T4 capacity of the thyroid gland

[1] 1.1662901 1.6883872 1.7643354 1.6416093 2.1387528 2.1379972 1.5389122 1.9342123 1.6258049 1.8479766 1.7873319 1.4665963

[13] 1.6287151 2.3374480 1.7689232 2.0158044 2.1043729 1.7723598 1.8181749 1.6396776 2.1689952 1.8564319 1.9320232 1.8597426

[25] 2.1999582 2.0261693 1.6053986 2.1888645 2.0655303 1.7392771 2.0951980 2.2277403 2.9376707 2.3114473 1.9416157 1.9144412

[37] 1.8570034 2.5080254 2.1221753 2.1360043 2.3190810 2.0433861 2.2591870 2.1291618 2.1013114 2.5085423 2.1713087 2.6646390

[49] 2.7167887 2.5060575 2.1152082 2.1476374 2.4520270 2.6718639 2.0733277 2.7982257 2.2604960 2.4393912 2.5604957 2.4110965

[61] 2.4978266 2.4719875 2.5421148 2.9357580 3.0671694 2.8281431 2.4039217 2.6764523 2.0262683 2.3171461 2.2102009 2.5918184

[73] 2.3729552 2.1995212 2.4228960 2.1516763 2.6217905 2.9274396 2.4883237 2.6712886 2.4334342 2.7810676 3.1652941 2.8886345

[85] 2.4786347 2.2808025 3.1043926 3.2172796 2.5481476 3.0011516 1.9315224 3.1813310 2.7836646 2.6597887 2.7823490 2.2719017

[97] 2.3955465 2.5307789 2.8302366 2.3649922 2.2953589 2.8205681 2.5287852 2.4509764 2.6248628 2.8991022 2.8307344 2.9394769

[109] 2.9500524 3.2272385 3.1992021 2.6229483 2.7024316 2.8120309 2.7123135 2.7721440 3.1026362 2.8244909 3.2105724 2.7025705

[121] 2.9870516 2.9985311 3.0424752 2.3435282 2.6518871 3.6053428 2.6004322 3.2661428 3.1249542 2.6323976 2.8429894 2.3375691

[133] 2.5798896 2.9304623 2.9428497 2.6885146 2.6686307 3.3995734 3.4431577 2.9988485 2.5945856 4.2188065 2.9377030 3.4837310

[145] 2.7099689 3.3235911 3.5213353 2.7716316 3.4986170 5.5930338 3.1584599 2.8035518 3.5218172 3.1437428 3.0738818 3.0270971

[157] 2.8766150 3.3938148 3.2198218 3.3159359 3.6963235 3.9862312 3.8072761 3.1799808

3.9004452 3.4732445 3.8480551 3.1983835

[169] 3.5232193 3.6731435 3.4935646 3.4684310 3.9711022 4.5994411 3.3930303 3.9693090

3.9693090 3.5861135 3.4239161 3.6298660

[181] 3.5228031 4.5330187 3.8054923 3.2841920 3.8558044 2.8960047 4.1713094 4.0649296

4.7067606 3.9044718 4.4149275 3.7670302

[193] 3.8461573 3.5686199 3.9559120 3.6494766 4.0673556 4.3738002 4.6063697 3.9563911

4.4933299 3.5890120 4.3838602 4.3838602

[205] 3.8427835 3.7271526 4.3916837 4.1339409 3.6681447 3.3479099 4.0800165 3.2183034

3.9930801 4.3000281 4.1183368 4.8239355

[217] 4.5871330 4.6570119 4.7501521 3.3530486 4.5949184 3.8828476 4.6115496 5.0537530

5.0983946 4.6841500 4.3973653 5.2405382

[229] 4.1202503 4.1641812 4.4039626 5.1379564 3.6717202 5.3559955 3.9118187 5.0343406

5.2384355 5.4482409 5.3990700 4.5225976

[241] 4.4524798 4.6761176 5.1366443 5.8451470 4.4751586 4.3677548 4.7973700 5.8901664

5.5974874 5.5489872 6.0668927 6.5847370

[253] 5.6494051 5.0712949 6.3201958 5.1469858 4.9777522 4.8823236 5.8510386 6.0715753

6.3151626 5.5865349 7.7299087 8.1220026

[265] 7.4890239 7.4890239 7.5410310 8.2691306 8.7934642 7.8929287 7.5573618 8.7087991

6.6353513 4.6708694 4.7600192 3.5439593

[277] 2.8485441 3.4889468 3.8934623 3.8480551 3.4196681 4.2095569 4.2364664 2.7642296

2.9377030 3.4867420 2.7865960 2.4463145

[289] 3.2891623 1.8839048 2.0116878 2.8244909 2.9735293 2.5986193 2.9921586 2.6965400

2.8281431 2.3797004 2.5559745 2.4348542

[301] 2.8767149 2.3340743 2.4341681 2.2000988 2.6438717 2.2786925 2.0340736 2.3114473

2.1870771 2.6306103 2.9543799 1.8369937

[313] 2.1851933 2.0307877 1.6414934 2.1610253 2.1542493 1.7346266 1.8762172 1.5164180

1.5378676 1.5260476 1.7512988 1.4665963

[325] 1.5991917 1.5687859 1.5736471 1.9997468 2.0495970 1.9721497 1.4089472 1.7315494

2.1387528 1.5336454 1.9004922 1.6416093

[337] 1.5366813 1.3519326 1.6189746 1.6046742 1.4986958 1.7643354 1.8024825 1.6236250

1.5438033 1.4216123 1.4315691 1.4391460

[349] 1.2849365 1.6883872 1.0932139 1.1490338 1.2629639 1.2831114 1.3409346 0.8838186

0.7363843

FT3 # serum FT3 concentration

[1] 5.30 6.80 5.60 4.80 3.40 5.70 4.55 5.20 5.78 5.00 6.70 4.60 5.20 6.70 5.30 5.10 5.19 3.60 5.10 4.70

5.20 5.00 5.80 5.59 4.30

[26] 4.59 4.30 4.00 4.80 5.10 4.40 5.40 5.80 4.80 4.90 4.80 5.00 5.01 5.17 5.60 5.10 5.20 4.80 6.43 5.00

5.50 5.60 5.32 5.60 5.60

[51] 5.00 4.87 5.50 5.50 5.30 5.10 5.60 4.80 5.40 4.70 4.70 4.60 6.20 5.41 5.90 5.00 5.50 5.20 4.11 5.40

5.70 4.20 4.60 4.60 4.59

[76] 5.00 6.10 3.38 4.80 5.30 5.40 5.60 6.11 5.21 5.23 4.80 3.91 5.10 4.77 4.96 4.30 5.17 5.40 4.53 5.90

4.83 5.10 5.88 4.40 5.00

[101] 4.70 4.90 5.19 5.50 5.32 5.70 4.70 5.30 4.18 6.10 4.60 4.78 5.30 4.60 4.65 6.11 4.50 3.73 5.08 5.50

6.00 5.20 4.50 4.80 4.90

[126] 5.30 5.20 5.20 5.07 4.60 5.00 5.43 4.80 4.30 4.50 5.30 4.45 4.80 5.40 4.90 5.38 4.60 4.70 4.60 4.50

4.40 4.80 4.85 4.99 6.30

[151] 4.99 4.60 4.80 4.30 4.70 4.90 5.32 5.00 4.80 5.10 5.65 5.80 4.41 4.27 4.40 4.20 4.98 5.10 5.57 5.65

4.50 4.90 5.00 5.20 5.30

[176] 4.70 5.50 4.50 5.40 5.60 5.60 6.21 4.90 5.10 4.70 4.15 5.40 4.50 4.93 5.20 4.90 4.80 5.50 4.70 5.30

4.49 4.99 5.60 4.80 5.00

[201] 5.26 5.30 5.00 5.10 4.90 4.80 5.80 4.35 4.40 4.96 4.70 4.60 5.30 5.28 5.75 5.43 5.10 3.68 4.29 4.60

5.00 4.40 4.50 5.20 4.30

[226] 4.90 5.50 5.20 4.80 4.80 3.70 5.30 4.40 5.21 4.70 4.90 5.20 6.00 4.90 5.44 5.51 4.70 5.00 5.51 4.35

5.30 5.94 5.10 5.30 4.17

[251] 5.00 5.10 5.27 4.40 4.79 4.90 5.57 5.08 5.30 5.30 4.90 4.67 6.35 4.90 4.68 5.04 5.12 5.97 5.01 5.10

4.70 4.50 4.70 4.95 4.58

[276] 4.98 4.70 4.90 5.00 4.98 5.00 4.90 5.70 4.40 4.90 4.43 5.00 4.70 5.80 3.60 4.40 3.73 5.30 4.60 5.82

5.10 5.00 4.90 3.68 4.90

[301] 4.80 7.30 5.90 6.20 4.30 4.50 4.90 4.80 4.40 4.90 5.60 4.69 5.30 4.80 4.10 4.20 5.00 5.44 4.80 4.90

4.50 4.80 5.15 4.60 5.20

```
[326] 4.40 5.50 5.40 4.60 5.40 4.76 5.20 3.40 5.04 5.67 4.80 5.00 4.80 5.40 6.20 5.26 5.60 6.09 4.68 5.10
4.89 5.00 5.10 5.00 6.80
[351] 4.50 4.90 4.70 5.00 4.50 4.30 4.50
# vectors of GT and FT3 are combined to a data frame cap1
# plot percentage change
p6c=ggplot() +
geom_point(aes(x = GT/3.43*100,y = FT3/5.04*100),data=cap1,colour = '#33ff00',size = 1.0) +
geom_smooth(aes(x = GT/3.43*100,y = FT3/5.04*100),data=cap1,colour = '#66ff00',size = 0.8,method =
'lm') +
geom_point(aes(x = GT/3.43*100,y = FT4/14.2*100),data=cap1,colour = '#0066ff',size = 0.8) +
geom_smooth(aes(x = GT/3.43*100,y = FT4/14.2*100),data=cap1,method = 'lm') +
scale_x_continuous(breaks = c(c(40,60,80,100,120,140)),limits = c(25,110)) +
scale_y_continuous(limits = c(50,150)) +
theme_classic() +
xlab(label = 'Estimate of FT4 Production Rate Constant (%)') +
ylab(label = 'FT4 FT3 (%)')
p6c

# system(10)      vary several parameters simultaneously
parRanges <- data.frame(min = c(0.8,0.2,0.1,0.1,0.2,0.2), max = c(1.2,0.3,0.4,0.2,0.8,0.8));
rownames(parRanges) <- c("k32","k423","k43","k42","s1","c2");
r1 <- sensRange(func=fs2,parms = k,parRange=parRanges,
sensvar=c("FT4","FT3","TSH","TRH"),num=1000);
# plot
p6d=ggplot() +
geom_point(aes(x = k32*100,y = FT3/1.41*100),data=r1,colour = '#33ff00',size = 0.5) +
geom_point(aes(x = k32*100,y = FT4/1.19*100),data=r1,colour = '#0066ff',size = 0.5) +
#geom_point(aes(x = k32*100,y = TSH/1.19*100),data=r1,size = 0.5) +
theme_classic() +
xlab(label='Estimate of FT4 Production Rate Constant (%)') +
ylab(label='FT4 FT3  (%)') +
```

ylim(50,150)

p6d

Draw Fig. 6e, using the model of Hoermann et al. 2020 plus code in Supplementary Material,

Hoermann R el al. Triiodothyronine secretion in early thyroid failure: The adaptive response of central feedforward control. Eur J Clin Invest. 2020;50:e13192. doi:10.1111/eci.13192

Draw Fig 6f, deidentified data were available from a clinical study by Hoermann et al. 2014.

Hoermann R, Midgley JEM, Giacobino A, Eckl WA, Wahl HG, Dietrich JW, Larisch R. Homeostatic equilibria between free thyroid hormones and pituitary thyrotropin are modulated by various influences including age, body mass index and treatment. Clin Endocrinol (Oxf). 2014;81:907-915. doi:10.1111/cen.12527

GTperc # percentage of maximum thyroid T4 secretory capacity

[1] 2584.07241 6719.11942 813.89394 229.86103 277.97148 72.27912 297.77916 178.40037

5180.20648 247.33286 247.33286

[12] 804.55332 523.95713 388.40759 251.69622 378.00580 1444.36407 269.57995 NA

546.75838 1076.03289 230.77479

[23] 509.38872 756.89685 608.82491 579.62364 467.39776 510.25890 189.96033 388.76723

368.84172 318.69341 607.96897

[34] 169.75302 3586.29679 195.19971 364.03134 299.25459 277.08622 1914.15230 282.60328

184.13397 4567.16429 222.73355

[45] 228.95259 211.46053 225.81294 82.13431 48.56683 121.41887 224.26323 76.38664

74.90857 41.49737 196.25711

[56] 92.13638 132.32487 100.30852 100.30852 142.82686 86.27420 161.15284 128.73386

122.06745 147.12665 64.02167

[67] 71.04822 56.97769 380.57726 211.71980 80.69298 226.24950 157.95822 190.59789

197.10724 61.93327 92.53538

[78] 45.94100 123.72803 114.08300 169.83620 59.78495 188.66116 225.99171 50.80620

146.67877 138.42594 143.43769

[89] 160.89986 130.61157 103.89836 85.68611 69.56985 118.81114 106.91375 106.91375

104.68372

fT3 # serum FT3 concentration

```
[1] 1.5 0.7 1.2 2.7 1.1 0.0 1.1 1.2 1.4 1.7 1.7 2.3 2.6 3.2 3.3 2.9 3.3 2.8 3.2 2.9 2.9 2.6 2.1 1.8 2.3 2.8 2.5
3.4 2.5 2.7 2.6 3.3
[33] 2.8 3.1 2.1 2.3 2.2 2.9 2.3 2.2 3.0 2.7 1.7 1.2 0.6 2.3 1.6 3.2 2.8 3.0 2.9 3.2 2.7 3.2 2.9 2.8 2.7 3.1 3.1
2.5 2.6 3.3 3.4 2.5
[65] 2.5 2.2 1.9 2.5 2.1 3.1 2.8 3.4 2.7 2.6 2.3 0.4 1.6 2.5 3.2 2.4 2.8 1.5 1.2 1.9 1.8 1.4 1.6 1.9 1.0 0.9 0.7
0.8 1.5 0.7 1.3 0.0
[97] 2.1
# vectors of GT and fT3 are combined into a data frame low2.
# plot
p6f=ggplot() +
geom_point(aes(x = GTperc,y = fT3/3.7*100),data=low2,colour = '#66ff00') +
geom_smooth(aes(x = GTperc,y = fT3/3.7*100),data=low2,colour = '#66ff00',method = 'lm') +
geom_point(aes(x = GTperc,y = fT4/14.9*100),data=low2,colour = '#3366ff') +
geom_smooth(aes(x = GTperc,y = fT4/14.9*100),data=low2,method = 'lm') +
theme_classic() +
xlab(label = 'Estimate of FT4 Production Rate Constant (%)')+
ylab(label = 'FT4 FT3 (%)') +
scale_x_continuous(limits = c(70,130)) +
ylim(0,150)
p6f
# Fig. 6, arrange panels
p6=p6a + plot_spacer() + p6c + p6d + plot_spacer() + p6f + plot_annotation(tag_levels = 'A') +
plot_layout(nrow=3, byrow=TRUE)
p6
```
